# Why the Long Face? The Mechanics of Mandibular Symphysis Proportions in Crocodiles

**DOI:** 10.1371/journal.pone.0053873

**Published:** 2013-01-16

**Authors:** Christopher W. Walmsley, Peter D. Smits, Michelle R. Quayle, Matthew R. McCurry, Heather S. Richards, Christopher C. Oldfield, Stephen Wroe, Phillip D. Clausen, Colin R. McHenry

**Affiliations:** 1 Department of Anatomy and Developmental Biology, School of Biomedical Sciences, Monash University, Melbourne, Victoria, Australia; 2 School of Engineering, University of Newcastle, Newcastle, New South Wales, Australia; 3 School of Biological Sciences, Monash University, Melbourne, Victoria, Australia; 4 Committee on Evolutionary Biology, University of Chicago, Chicago, Illinois, United States of America; 5 School of Biological, Environmental and Earth Sciences, University of New South Wales, Sydney, New South Wales, Australia; University of Zurich, Switzerland

## Abstract

**Background:**

Crocodilians exhibit a spectrum of rostral shape from long snouted (longirostrine), through to short snouted (brevirostrine) morphologies. The proportional length of the mandibular symphysis correlates consistently with rostral shape, forming as much as 50% of the mandible’s length in longirostrine forms, but 10% in brevirostrine crocodilians. Here we analyse the structural consequences of an elongate mandibular symphysis in relation to feeding behaviours.

**Methods/Principal Findings:**

Simple beam and high resolution Finite Element (FE) models of seven species of crocodile were analysed under loads simulating biting, shaking and twisting. Using beam theory, we statistically compared multiple hypotheses of which morphological variables should control the biomechanical response. Brevi- and mesorostrine morphologies were found to consistently outperform longirostrine types when subject to equivalent biting, shaking and twisting loads. The best predictors of performance for biting and twisting loads in FE models were overall length and symphyseal length respectively; for shaking loads symphyseal length and a multivariate measurement of shape (PC1– which is strongly but not exclusively correlated with symphyseal length) were equally good predictors. Linear measurements were better predictors than multivariate measurements of shape in biting and twisting loads. For both biting and shaking loads but not for twisting, simple beam models agree with best performance predictors in FE models.

**Conclusions/Significance:**

Combining beam and FE modelling allows *a priori* hypotheses about the importance of morphological traits on biomechanics to be statistically tested. Short mandibular symphyses perform well under loads used for feeding upon large prey, but elongate symphyses incur high strains under equivalent loads, underlining the structural constraints to prey size in the longirostrine morphotype. The biomechanics of the crocodilian mandible are largely consistent with beam theory and can be predicted from simple morphological measurements, suggesting that crocodilians are a useful model for investigating the palaeobiomechanics of other aquatic tetrapods.

## Introduction

**Figure 1:**
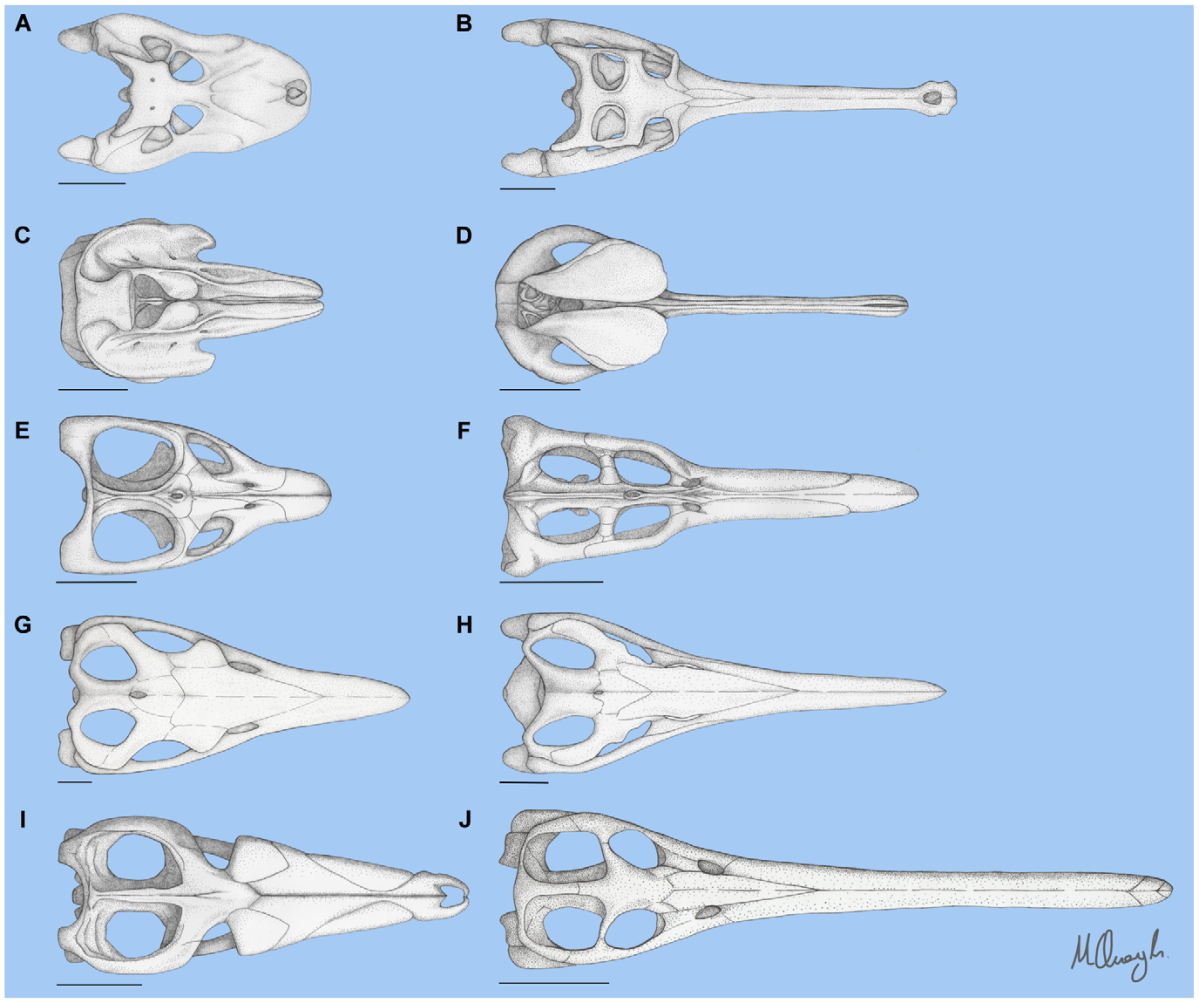
Spectrum of rostral proportions in marine tetrapods. Dorsal view of various skulls, showing the spectrum of rostral proportions in (from top) crocodilians, odontocetes, plesiosaurs, ichthyosaurs and thalattosuchians. Skulls are resized to equivalent width at the back of the skull and for each group longirostrine taxa are on the right, brevirostrine on the left. Taxa shown are *Caiman latirostris* (A), *Gavialis gangeticus* (B), *Feresa attenuata* (C), *Platanista gangetica* (D), *Leptocleidus capensis* (E), *Dolichorhynchops osborni* (F), *Temnodontosaurus eurycephalus* (G), *Ophthalmosaurus icenicus* (H), *Suchodus brachyrhynchus* (I), *Steneosaurus gracilirostris* (J). Scale bars = 10 cm. Based on photos by CRM of specimen BMNH 86.10.4.2 (A), BMNH 1935.6.4.1 (B), BMNH 1897.6.30.1 (C) and USNM 504917 (D), after Cruichshank [60] (E), after O’Keefe [61] (F) based on fossil specimen BMNH R1157 illustrated by Owen [62] (G), after Motani [63] (H), after Andrews [64](I), after Mueller-Töwe [65](J).

Large aquatic predators operate in a physical environment that has driven remarkable morphological convergence, notably the independent evolution of a tunniform body form in ichthyosaurs (reptiles), lamnids (sharks), thunnids (bony fish) and odontocetes (mammals) [1], [2], [3], [4], [5]. In addition to swimming, feeding behaviour operates under strong constraints based on the fundamental fluid dynamics of water that apply to ram, filter, and suction feeders [6]. For ram feeding, a spectrum of skull morphology runs from elongate, narrow ‘pincer’ jaws (‘longirostrine’) to shorter, more robust jaws (‘brevirostrine’). This spectrum of jaw morphologies exists in a wide range of secondarily aquatic amniotes, including crocodilians, ichthyosaurs, plesiosaurs, and odontocetes (Figure 1).

**Figure 2:**
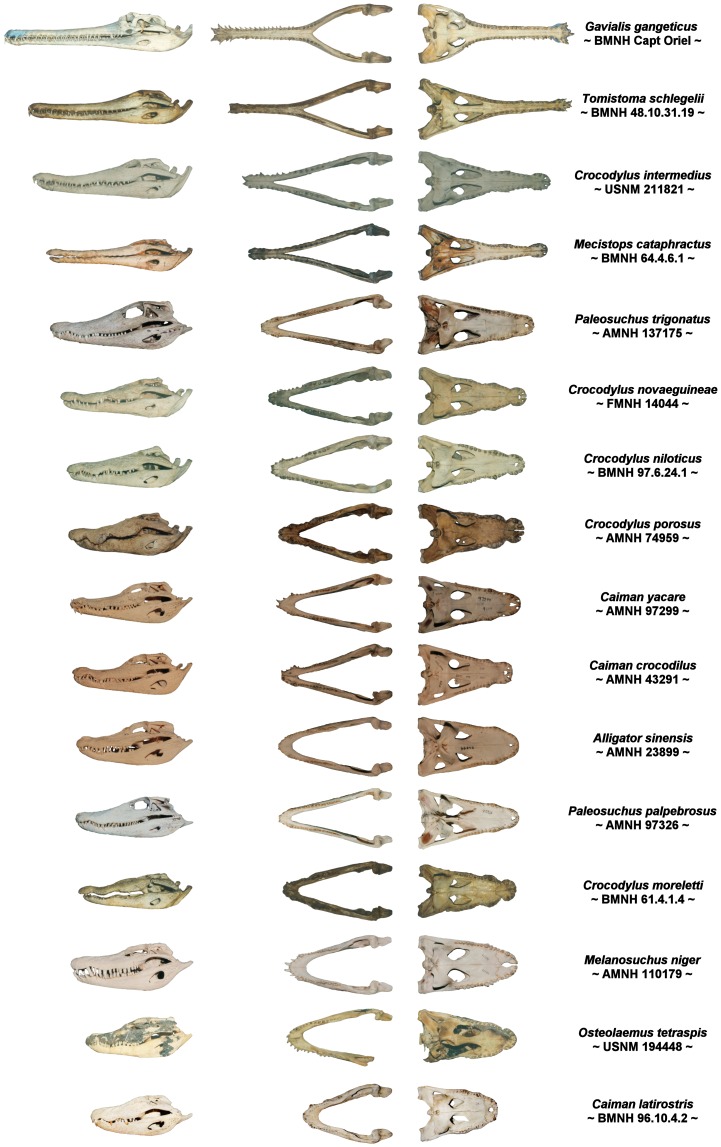
Range of skull shape in crocodilians. Specimens are scaled to approximately the same width and arranged from most longirostrine to most brevirostrine. Left: cranium and mandible in lateral view, Centre left: dorsal view of mandible, Centre right: Cranium in ventral view, Right: species name and specimen number.

Among the 24 extant species of crocodilians, head shape ranges from the hyper-long snouted animals such as the gharial *(Gavialis gangeticus)* and false gharial *(Tomistoma schlegelii),* through to broad-snouted brevirostrine taxa such as the spectacled caiman *(Caiman crocodilus)* and dwarf crocodile *(Osteolaemus tetraspis)* (Figure 2). Rostral shape correlates consistently with feeding behaviour; long slender-snouted crocodilians tend to concentrate on small, agile, aquatic prey (fish), whilst shorter and more robust-snouted animals often take much larger prey [5], [7], [8]. The Gharial *(Gavialis gangeticus)* is the longest snouted form and is described as a specialist fish eater [7], [9], whilst the saltwater *(Crocodylus porosus)* and Nile *(C. niloticus)* crocodiles have shorter, more robust snouts and are capable of taking terrestrial prey much larger than themselves [10]. This relationship between head shape and diet has been considered reliable enough to serve as a basis to infer diet in fossil species of marine reptiles and mammals [2], [5], [11].

**Figure 3:**
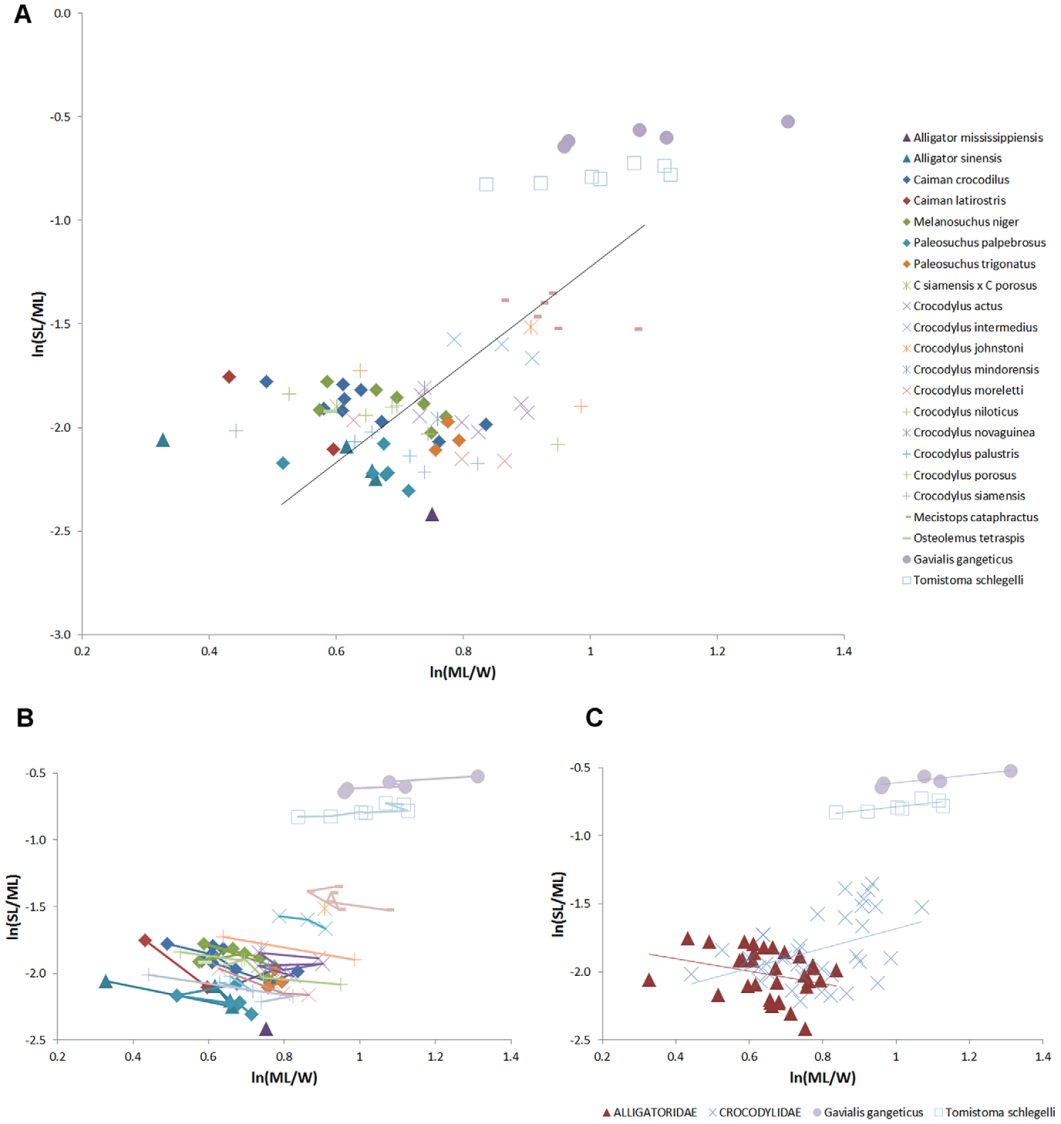
Mandibular symphysis length vs mandible length in extant crocodilians. X axis plots the ratio of mandibular length to width, giving a size-controlled proxy for the spectrum of brevisrostral to longirostral morphology. Y axis is the proportion of symphyseal length to mandibular length. Values shown are natural logarithms. (A), data for 82 specimens of crocodilian, data measured from photographs of museum skulls; regression line is based upon mean values for each species. (B), data points as for (A), with data points ordered by width in each species and connected by lines. In effect, this plot shows the allometric trajectory of ML/W for each species, with the smallest animals on the right and largest on the left of each species plot; i.e. as animals increase in size, head width increases as a proportion of head length. Within each species, the symphyseal length (as a proportion of mandible length) remains consistent. (C), Regression lines for alligatorids, non-tomistomine crocodylids, *Gavialis*, and *Tomistoma*.

Longirostrine aquatic predators consistently have an elongated mandibular symphysis, which in longirostrine crocodilians such as *Gavialis* and *Tomistoma* makes up half the length of the lower jaw. In general, longirostrine taxa have proportionally longer mandibular symphyses than do mesorostrine or brevirostrine relatives (Figures 2 and 3). As the longirostrine condition correlates with a preference for small agile prey (e.g. fish), an elongate symphysis can therefore act as a proxy for feeding ecology in some extinct groups [11]. The presence of elongated mandibular symphyses in longirostrine species in many unrelated groups suggests possible physical constraints on prey capture. The spectrum of jaw morphology in crocodilians has been interpreted as the functional trade-off between hydrodynamic agility and strength, with longirostrine skulls reflecting a low drag-high speed morphotype suited for capturing small agile prey, and meso- to brevirostrine skulls being low speed-high strength jaws better suited for killing and processing slower but larger or harder foods [5], [7], [8], [12]. In longirostrine forms, the elongated jaws provide extra reach and higher tip velocity, factors which likely contribute to success rates of capturing small agile prey. However, the rapid sideways sweeping of the jaws during feeding incurs high drag, a cost that increases quadratically with snout length for a given profile [8], and the reduced height and width of the jaws in longirostrine taxa may serve to minimise pressure and skin drag respectively, especially in the anterior portion of the jaw. Additionally, the reduction of rostral width and height in longirostrine crocodilians may reduce angular momentum and mass moment of inertia (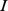) of the snout, decreasing the energy required to accelerate the jaws towards prey (which also increases the acceleration possible for a given muscular effort); it may also be a means of minimising drag incurred by the jaw during rapid adduction. Reduced distal mass is especially important for rapid adduction or sideways movements of longirostrine snout, because 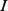 increases with the square of the distance of a unit of mass from the centre of rotation. In the upper jaw, the anterior snout has an almost tubular section and this is mirrored by the symphyseal part of the lower jaw in longirostrine crocodilians; the formation of an elongate symphysis seems to be a configuration allowing a minimal diameter of the mandible, and can be explained by hydrodynamic and/or energetic criteria.

If an elongate mandibular symphysis increases streamlining/energy efficiency, why is it not a consistent feature of all crocodilian mandibles? Why do forms with shorter rostra lack a long symphysis? While the longirostrine form is streamlined and is efficient for capturing small, agile aquatic prey, it is not strong or well suited to the loads that result from feeding on large prey [8]. In crocodilians that feed on large prey, the snout is shorter, broader, and usually taller in section than longirostrine forms; this shape is better for resisting high loads during feeding and is the defining characteristic of meso- and brevirostrine taxa [8]. Although the structural consequences of this morphology have been explored for the upper jaw, those for the lower jaw have received less attention [7], [8]. If an elongate symphysis is the most effective morphology for reducing the drag incurred and/or increasing the rate of acceleration of the anterior part of the mandible during a rapid lateral sweep, then the absence of an elongate symphysis in meso- and brevirostrine taxa may be enforced by structural mechanics; i.e. an elongate symphysis decreases the strength of the mandible.

### Theoretical Framework

#### Biomechanics of processing large prey for aquatic predators

The mechanics of feeding upon large prey in water have been detailed by Taylor [5] and are summarised here. For predators that feed on prey that are too large to be swallowed whole, rendering prey into bite-sized chunks is an important component of feeding behaviour. Terrestrial predators can use the weight of the prey to restrain it whilst the predator rips off chunks; the predator’s forelimbs can help secure the carcass, whilst shearing dentition produces the forces required to reduce prey. Aquatic predators, however, are unable to use the prey’s weight as an anchor because the predator cannot brace against the ground (both predator and prey are effectively weightless in water), and as forelimbs are often modified for aquatic locomotion these cannot be used to restrain prey. As a result, the aquatic predators often use vigorous shaking of the prey, provided the prey is small enough to be held clear of the water. When the prey is too large to shake, its inertia is used to anchor it whilst the predator spins rapidly around its own long axis, generating shear forces that twist chunks off the carcass [13]. Shake and twist feeding are also used to subdue prey after capture, with the use of twist feeding in crocodilians underlying the infamous ‘death roll’.

#### ‘Armchair predictions’: argument from principles of beam theory

**Figure 4:**
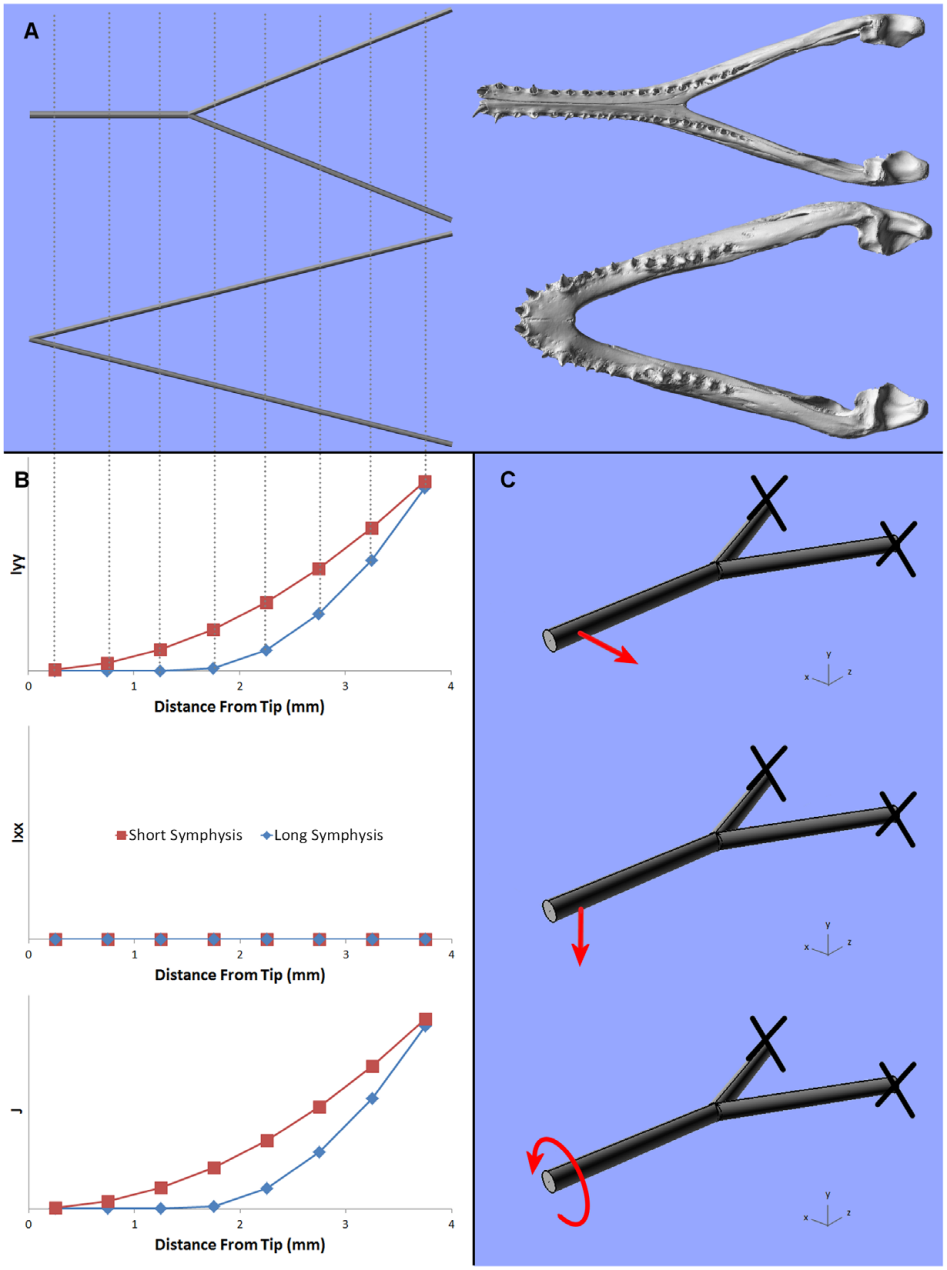
Second moments of area for beam models. Second moments of area correspond to the geometry of long and short symphysis crocodilians. (A) shows the beam approximation of mandibles with long and short symphyseal lengths. (B) shows the change in second moment of area (length^4^) for long and short symphyseal beam models; these were calculated at discrete locations from the tip (anterior) of each mandible, as a conceptual illustration of the differences in second moments of area between the two morphologies. Corresponding locations are shown with dotted lines and the Y axis is a uniform arbitrary scale throughout. (C) shows (from top) the loading regimes associated with *shaking*, *biting* and *twisting*; where red arrows represent forces and black crosses represent restraints.

In crocodilians that feed on large prey (too large to be swallowed whole), the skull must be capable of withstanding diverse loads: (1) straightforward adduction of the jaws (‘*biting’*), (2) vigorous lateral shaking of the head with the prey held in the jaws (‘*shaking’*), and (3) rapid roll of the predator’s whole body about the longitudinal axis, with the prey held in the jaws (‘*twisting’*). How these loads interact with symphyseal length can be explored based on beam theory. The mandible can be viewed as a ‘Y’ shaped beam configuration with uniform sections and X, Y, and Z axes representing the transverse, dorso-ventral, and longitudinal directions respectively (Figure 4). Beam theory predicts that during *biting* the mandible will behave as a cantilevered beam loaded in the dorso-ventral (Y) direction. For a given section, the mechanical response will depend only on the length of the whole mandible; the proportion of the mandible that is formed by the symphysis will not affect the area moment of inertia in the dorso-ventral direction (Ixx, about the horizontal x axis), and so symphyseal length is irrelevant. In *shaking*, the mandible acts as a cantilevered beam that is loaded laterally (X axis) at its anterior end and fixed posteriorly; its mechanics will be influenced by both the length of the beam and by the moment of inertia in the lateral direction (Iyy, about the vertical axis). Symphyseal length (SL) does affect Iyy; a longer SL means a reduced Iyy, with a change in Iyy at the junction of the symphysis with the rami. Under *twisting* loads, the crocodile skull is expected to act as a tapered cylinder (i.e. a cone, an efficient shape for torsional loads), and the mandible will be a partial cone; the mechanics should depend primarily on the polar moment of area (J), and as increased SL reduces J then SL is expected to affect the mechanical performance.

#### Methodological aspects

Skulls are far more complex than beams, which presents significant challenges for analyses of cranial mechanics. While some studies have successfully applied beam theory to generate insights into the functional aspects of cranial shape variation [7], [14], [15], recent focus has been on the use of Finite Element Analysis (FEA) of high resolution meshes to describe the mechanical response of complex skull geometries to the loads incurred during feeding behaviour [16], [17]. Whilst FEA offers many advantages for biomechanical analysis, the gap between the high accuracy of the FE models and the simple geometry explained by beam theory has meant that the results of high resolution biological FEA are rarely discussed with reference to underlying mechanical principles such as beam theory. This lack of a theoretical context means that the analyses do not attempt to test hypotheses of structure/function relationships constructed *a priori*, but are instead used to describe *post-hoc* patterns of variation from which underlying generalities might be elucidated. Whilst *post-hoc* approaches are valid and often necessitated by the complexity of biological datasets, and are an important means of generating hypotheses, *a priori* approaches have the capacity to test hypotheses.

An approach that uses beam modelling and high resolution FEA combines the strengths of both methods [18]. Beam modelling requires an explicit hypothesis of the aspects of morphology that are considered to be of the highest biomechanical importance. High resolution finite element (hi-res FE) modelling describes the complex mechanical behaviour of actual morphology, and allows the explanatory power of the beam models to be evaluated quantitatively. If the beam models are found to describe, even qualitatively, the pattern of variation in mechanical performance between morphologies, then they are useful approximations of reality, and aspects of morphology they encapsulate may be most important to the performance of the biological structure. Small discrepancies between FEA and analytical results from beam theory (as with CT cross sections) are informative about the influence of factors such as mesh and geometry resolution, and material properties, on both methods. Conversely, a large discrepancy between beam and hi-res FE models indicates that the complexity of the biological structure overwhelms the capacity for analysis using beam theory, and/or the aspects of shape that determine mechanical behaviour have not been captured in the beam model.

#### Aims

Here we explore the correlation between head length and symphyseal length in crocodilians using beam theory and FEA. Building from the assumption (based upon theory but yet to be demonstrated empirically) that the dynamics of a rapid lateral sweep of the jaws during prey capture selects for a narrow rostrum and an elongate mandibular symphysis, we hypothesise that shorter symphyses of meso- and brevirostrine crocodilians are selected for by the mechanics of the *shaking* and *twisting* behaviours used in feeding on large prey, but not by the mechanics of *biting* (jaw adduction).

Implicit in the above hypothesis is the assumption that the biomechanics of the crocodilian mandible can be elucidated using beam theory; a secondary aim here is to quantify the extent to which that assumption is valid. For this, we used the following criteria; if the pattern of variation in the mechanical performance of the actual mandibles (as modelled in hi-res FEA) correlates best with the linear morphological variable predicted by beam theory, then the biomechanics of the mandibles conform with the principles of beam theory. In contrast, if the pattern of variation in the hi-res FE dataset correlates better with another variable (whether a linear measurement or a metric of shape) then the beam models do not explain the biomechanics of the actual rostrum, and the mechanics of complex biological structures resists explanation using fundamental principles.

Our approach is to:

1. Explore the mechanics of beam models of the mandible under *biting*, *shaking*, and *twisting* loads in relation to a number of simple variables.
2. Compile a comparative dataset, based upon CT scans of several crocodilian species that between them show a spectrum of symphyseal length relative to mandibular length.
3. Using Finite Element software, construct a set of ‘simple’ (beam) and ‘complex’ (hi-res FE) models of each specimen, which are then analysed under simulated *biting*, *shaking*, and *twisting* loads.
4. The results from this modelling will be analysed to evaluate the specific hypotheses:

Hypotheses:

- Strain in beam models will correlate with mandibular length under *biting*, but with symphyseal length under *shaking* and *twisting*.
- Similarly, strain in complex FE models of crocodilian mandibles will correlate with mandibular length under *biting*, but with symphyseal length under *shaking* and *twisting*.
- The crocodilian mandible behaves as a beam, i.e. the simple variables that best explain variation in strain between beam models will also best explain variation in strain between complex FE models.

## Methods

### Specimens, Scans, and Image Processing

**Figure 5:**
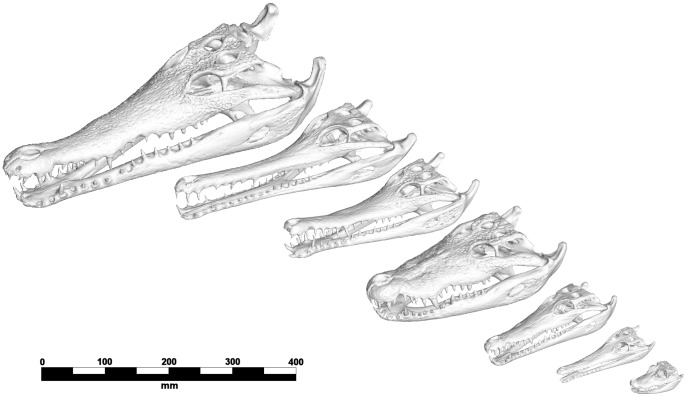
Specimen used in this study. From top left: *Crocodylus intermedius, Tomistoma schlegelii, Mecistops cataphractus, Crocodylus moreletii, Crocodylus novaeguineae, Crocodylus johnstoni, Osteolaemus tetraspis.*

Analysis was based upon seven species of crocodilian species spanning a large range of mandible morphology and symphyseal length (Table 1 and Figure 5). Models were constructed from CT scan data; five specimens were scanned at the University of Texas Digital Morphology Laboratories, one at the Newcastle Mater Hospital, and one at the US National Museum. Although scan settings are not identical for the different specimens, we did not have the opportunity to scan specimens upon multiple scanners and for the purposes of the present study we assume that the source of the scan does not affect the subsequent modelling results.

**Table 1:** Specimen scan information.

Species	Specimen	Scanned at	Scanned on
*O. tetraspis*	FMNH 98936	UT	UT High-Res X-Ray CT
*C. moreletii*	TMM M-4980	UT	UT High-Res X-Ray CT
*C. novaeguineae*	AM R24446	NMH	Toshiba Aquilion 64
*C. intermedius*	USNM	USNM	Siemens Definition AS+
*C. johnstoni*	TMM M-6807	UT	UT High-Res X-Ray CT
*M. cataphractus*	TMM M-3529	UT	UT High-Res X-Ray CT
*T. schlegelii*	TMM M-6342	UT	UT High-Res X-Ray CT

**Figure 6:**
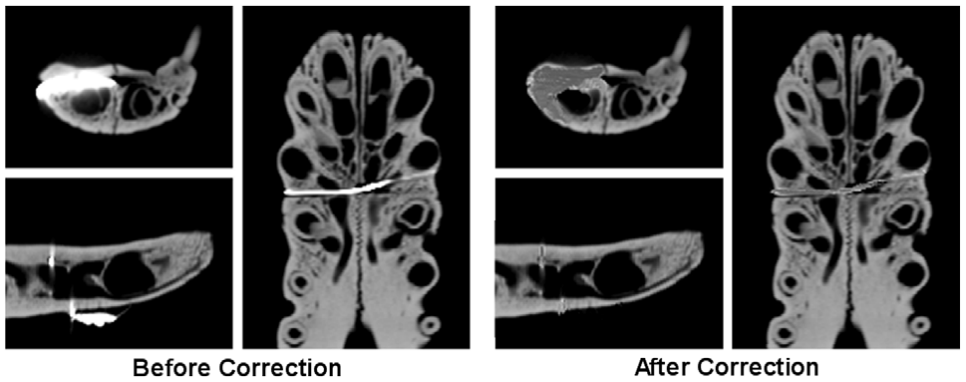
Manual correction of diffraction artefacts in *Crocodylus intermedius* scan. Left: scan data before correction. Right: scan data after correction. See text for explanation.

Processing of the CT data was performed in MIMICS v11 (MATERIALISE, Belgium). For each specimen, the skull and mandible were segmented separately and converted to 3D isosurface models. Image segmentation was largely straightforward, with the exception of the *Crocodylus intermedius* scan; this specimen had wire embedded in several positions within the mandible, resulting in refraction artefacts in the CT data; the affected slices were manually processed in a bitmap editor (Paintshop Pro v8, JASC) to improve image quality and reduce the influence of the artefacts (Figure 6).

**Figure 7:**
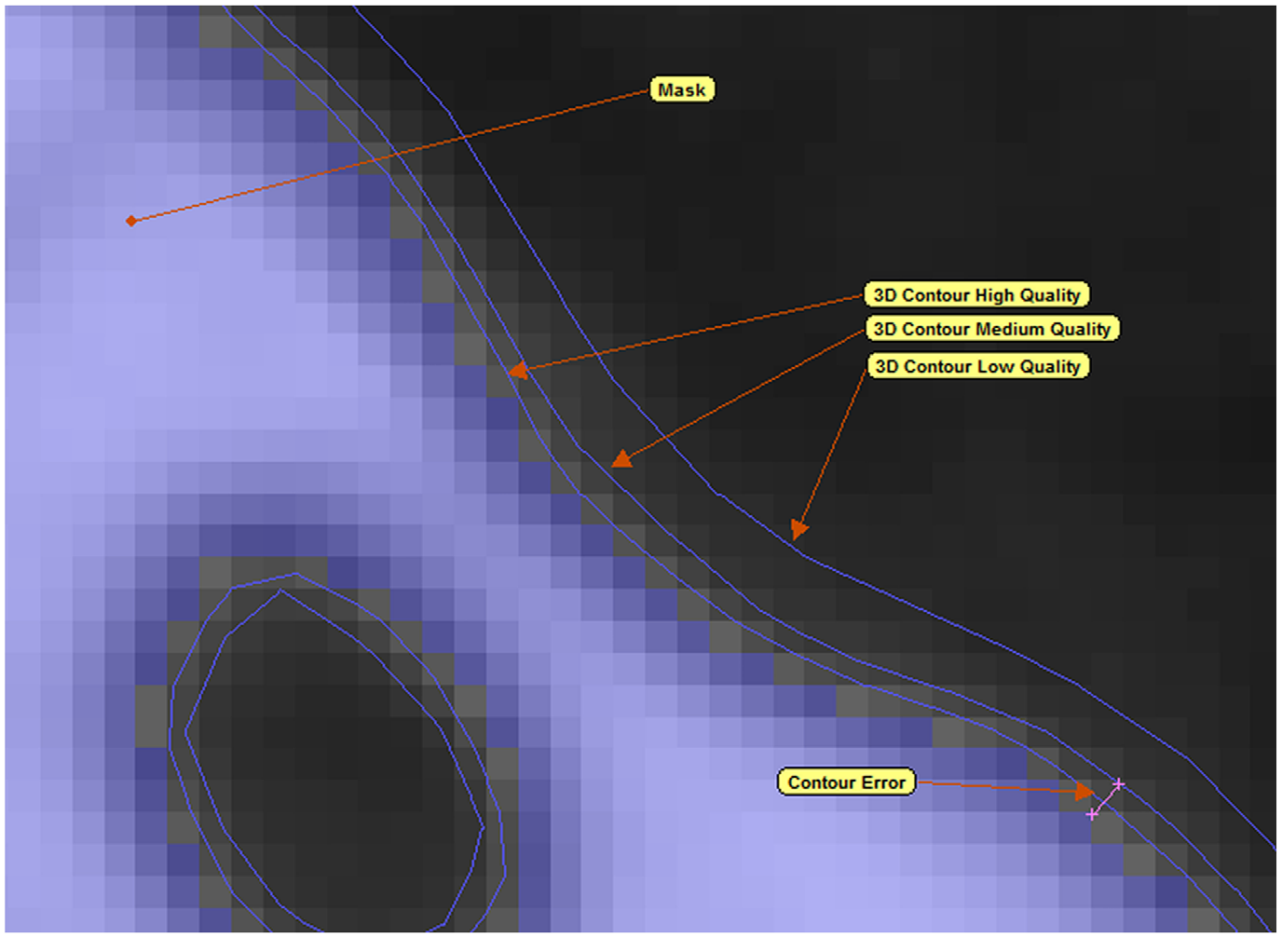
Quality of isosurface models and error quantification. The mask (shown in blue) represents the segmented/selected voxels that will be used to create isosurfaces. The three different contour qualities represent the 3D approximation of the mask and will form the isosurface. Contour error is the measured distance between the isosurface contour and the mask it was generated from (lower left of image).

Isosurface 3D models of segmented data can be made at low, medium, or high ‘quality’ - these settings exchange accuracy with computational requirements (Figure 7). The accuracy of the isosurface model was measured by averaging the difference between isosurface and segmentation mask diameters as measured at 10 locations on the mandible and cranium. For different specimens, a given quality setting gave a wide range of isosurface accuracy values (‘Average Contour Error’ in Table 2); presumably because of the different scan resolutions between specimens. For the final isosurface that formed the basis for the FE model, we standardised the level of accuracy by using the quality setting that gave a contour error between 0.05 and 0.1% of mandible length.

**Table 2:** Calculation and standardisation of error in the 3D models.

Taxon	3D Quality	Mimics MandibularLength (mm)	Average ContourError (mm)	Error% of MandibularLength
*O. tetraspis*	Medium	94.901	0.075	0.08%
*C. moreletii*	Medium	329.17	0.266	0.08%
*C. novaeguineae*	High	214.44	0.1595	0.07%
*C. intermedius*	High	581.94	0.3435	0.06%
*C. johnstoni*	High	171.12	0.146	0.09%
*M. cataphractus*	Medium	382.81	0.2315	0.06%
*T. schlegelii*	Medium	402.13	0.217	0.05%

**Figure 8:**
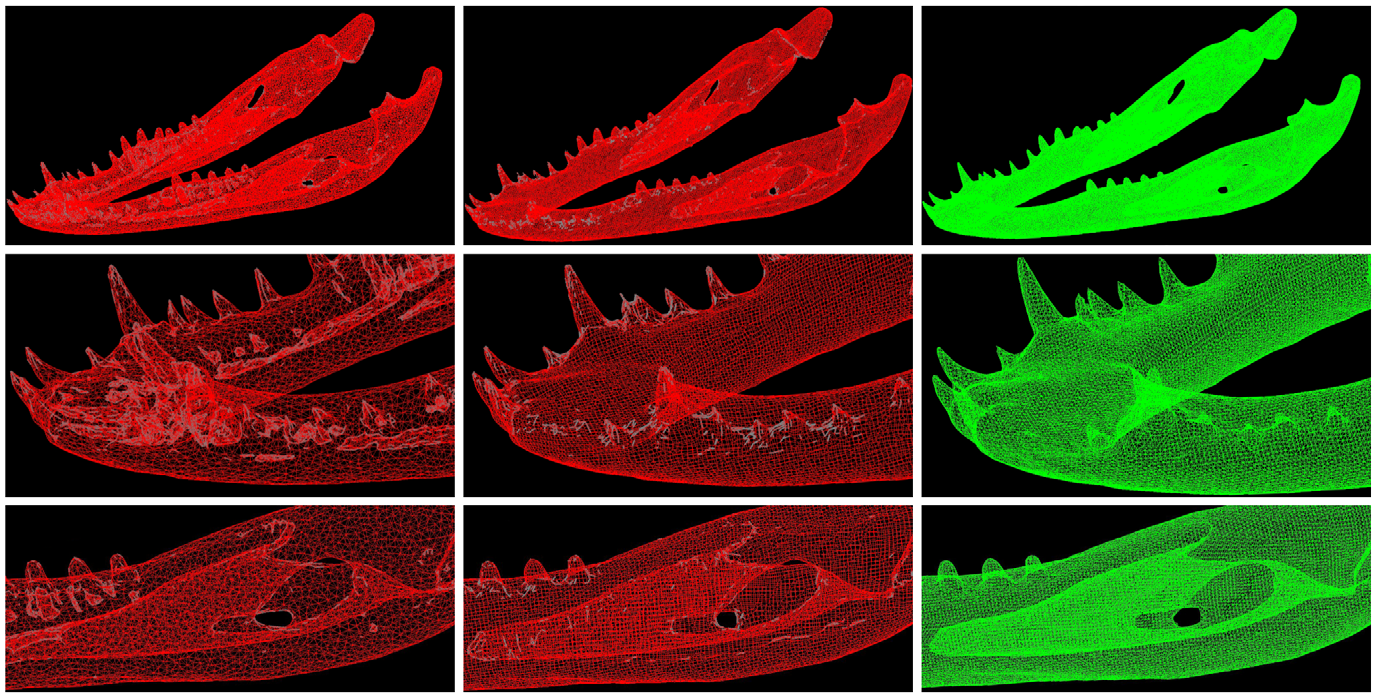
Mesh optimisation and solid mesh generation. Mesh optimisation and solid mesh generation was performed using Harpoon (SHARC). The left images show the complex internal geometry captured from isosurface generation. The middle column shows removal of complex internal geometry whilst still retaining important geometrical features. Images at right show the final solid mesh.

Isosurfaces were exported as STL (Stereolithography) files – a surface mesh comprising triangles. Surface meshes were used for morphometric analysis (see below) and formed the foundation upon which suitable FEA solid meshes were generated using Harpoon (SHARC). Surface meshes were optimised to remove unwanted internal geometry (Figure 8) and to control the resolution of the final ‘tetrahedral’ solid mesh. For each specimen, solid mesh resolution was set such that the number of tetrahedral elements in the cranium was approximately 1.5 million. The mandible was then meshed such that the average size of tetrahedral elements was approximately the same as the cranium, yielding 2.5 million tetrahedra (+/−10%) (Table 3) for the cranium and mandible combined.

**Table 3:** Mesh resolution for ‘complex’ FE models.

	Number of Elements		
Taxon	Cranium	Mandible	Total
*Osteolaemus tetraspis*	1523489	775787	2299276
*Crocodylus moreletii*	1564655	1028342	2592997
*Crocodylus novaeguineae*	1506529	804416	2310945
*Crocodylus intermedius*	1483496	999305	2482801
*Crocodylus johnstoni*	1504342	946364	2450706
*Mecistops cataphractus*	1493841	1146206	2640047
*Tomistoma schlegelii*	1516343	1105077	2621420

### Morphometrics

**Figure 9:**
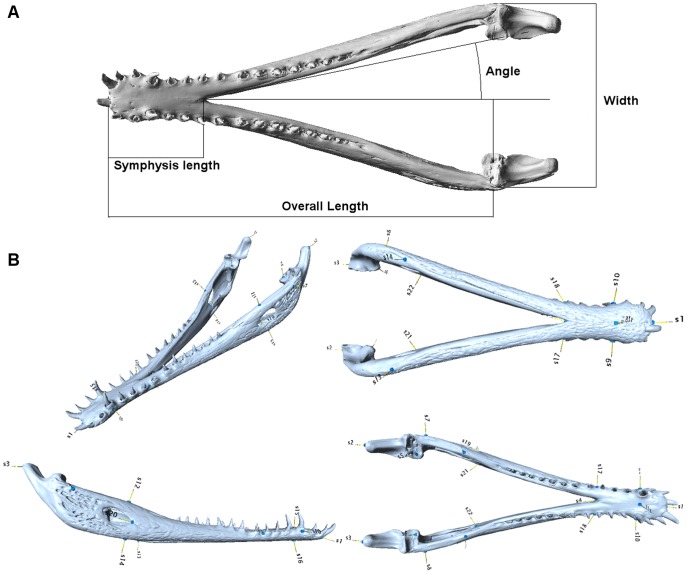
Linear measurements and landmarks for mandible. (A), linear measurements of mandible; (B), landmark locations. See text for explanation.

We used linear measurements and landmark coordinates from each mandible in order to quantify shape. Linear measurements comprised overall length (L), symphyseal length (SL), width (W), and inter-rami angle (A), and were taken from the STL files within Rhino (McNeel - [19]) (Figure 9A). Linear measurements were corrected for size using skull (cranium+mandible) volume. For multivariate quantification of shape, the surface mesh was imported into Landmark [20] as.PLY files and 22 landmarks were defined. (Table 4 and Figure 9B). These landmark locations were then exported to Morphologika v2.5 [21], where procrustes superimposition and principal component analysis were undertaken.

**Table 4:** Landmark characterisation.

Landmark Number	Location
S1	Anterior of jaw (origin) – midline
S2	Posterior apex of mandible (retro-articular process) – left
S3	As above – right
S4	Posterior apex of symphysis (dorsal margin) – midline
S5	Dorsal apex of ant-medial rim of joint socket (used to align trans. axis) – left
S6	As above – right
S7	Lateral apex of ramus at surangular – left
S8	As above – right
S9	Lateral apex of alveolus at widest part of symphysis – left
S10	As above – right
S11	Dorsal apex of coronoid process – left
S12	As above – left
S13	Ventral apex of ventral surface of ramus, directly ventral to coronid process (S11) – left
S14	As above – right
S15	Dorsal apex of symphysis at widest point (i.e. between S7 and S8) – midline
S16	Ventral apex of symphsysis – approx ventral to S15 – midline
S17	Ventral to the posterior apex of the symphysis – left
S18	Ventral to the posterior apex of the symphysis – right
S19	Anterior point of the external mandibular fenestrae – left
S20	Anterior point of the external mandibular fenestrae – right
S21	Anterior point of the adductor fossa – left

### Structural Modelling

We used the Finite Element Analysis package Strand7 [22] for analysis of beam and complex models. Beam models were constructed from 3 elements, whilst the complex (hi-res FE) models of the mandibles ranged between 0.75 and 1.15 million elements.

**Figure 10:**
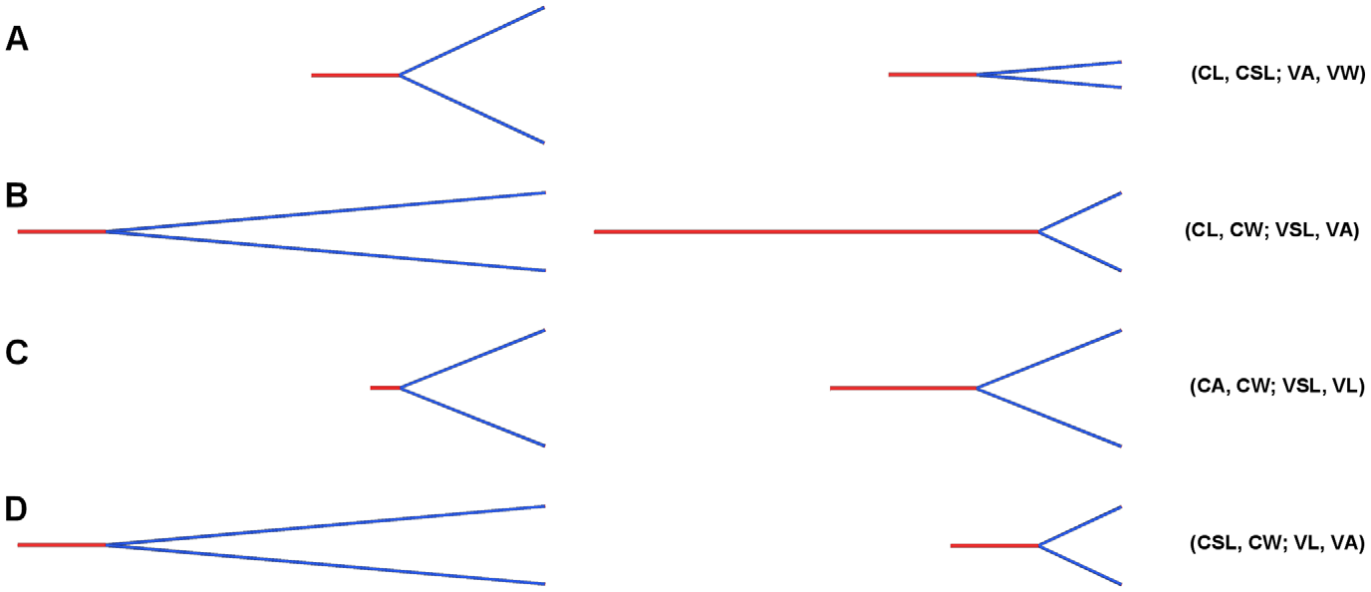
Variations for beam models #1. Model variations used to explore relationship between strain and linear variables in the first set of beam models. Abbreviations are defined as follows: (CL, CSL; VA, VW) – Constant length and symphyseal length, variable angle and width. (CL, CW; VSL, VA) – Constant length and width, variable symphyseal length and angle. (CA, CW; VSL, VL) – Constant angle and width, variable symphyseal length and length. (CSL, CW; VL, VA) – Constant symphyseal length and width, variable length and angle.

Three sets of models were produced. The first set (beam models #1) explored the effects of four linear variables - overall length (‘Length’, L), symphyseal length (SL), width (W), and inter- rami angle (‘Angle’, A) - upon stresses in the beam model representing the mandible. Within a mandible, these measurements co-vary and so their effects cannot be explored independently of each other. We therefore created four sets of models, within which two of the measurements were kept constant while two co-varied (Figure 10);

1. constant length and symphyseal length, variable angle and width (CL,CSL;VA, VW)
2. constant length and width, variable symphyseal length and angle (CL, CW; VSL, VA)
3. constant angle and width, variable symphyseal length and length (CA, CW; VSL, VL)
4. constant symphyseal length and width, variable length and angle (CSL, CW; VL, VA)

**Figure 11:**
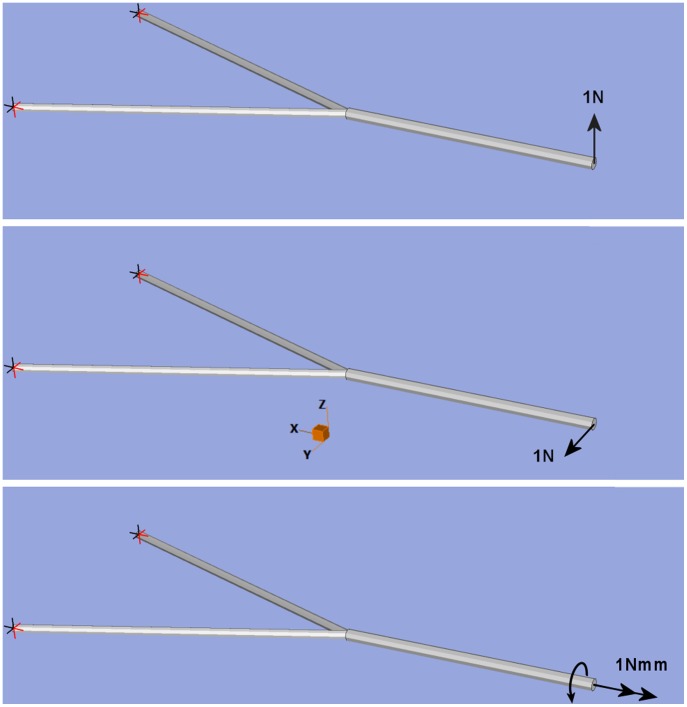
Beam models showing axes, restraints and loads. From top; shows loads and restraints for *biting, shaking* and *twisting* respectively. In all three cases models are fully restrained (rotation and translation) at the most posterior points of the beam model. Loads are all placed at the most anterior point of the beam model.

Beam dimensions are given in Table 5; the models were fully restrained at the two nodes at the rear (i.e. no rotations or translations in any axis), and a load was applied to the node at the front of the model (Figure 11). For the bite and shake load a 1 N force was applied in the X and Y axis respectively; for the twist a moment of 1 Nmm was applied in the XY plane.

**Table 5:** Dimensions for beam models #1.

Model Variation	Angle (Deg)	Overall Length (mm)	Symphyseal Length (mm)	Width (mm)
CL, CSL; VA, VW	10	4.0	1.5	0.4
	20	4.0	1.5	0.9
	30	4.0	1.5	1.3
	40	4.0	1.5	1.8
	50	4.0	1.5	2.3
CL, CW; VSL, VA	10	9.1	1.5	1.3
	20	9.1	5.3	1.3
	30	9.1	6.6	1.3
	40	9.1	7.2	1.3
	50	9.1	7.6	1.3
CA, CW; VSL, VL	44	3.0	0.5	2.0
	44	3.5	1.0	2.0
	44	4.0	1.5	2.0
	44	4.5	2.0	2.0
	44	5.0	2.5	2.0
CSL, CW; VL, VA	10	9.1	1.5	1.3
	20	5.3	1.5	1.3
	30	4.0	1.5	1.3
	40	3.3	1.5	1.3
	50	2.9	1.5	1.3

For all models symphyseal beam diameter = 0.069054 mm and Rami beam diameter = 0.05 mm.

Model variation abbreviations are defined as follows:

(CL, CSL; VA, VW) – Constant length and symphyseal length, variable angle and width.

(CL, CW; VSL, VA) – Constant length and width, variable symphyseal length and angle.

(CA, CW; VSL, VL) – Constant angle and width, variable symphyseal length and length.

(CSL, CW; VL, VA) – Constant symphyseal length and width, variable length and angle.

The second set of beam models (beam models #2) used a similar construction, but dimensions were adjusted to capture the corresponding geometry of the hi-res models (Table 6). This allows direct comparison between the results of the hi-res FE mandible models and beam modelling.

**Table 6:** Dimensions for beam models #2.

Beam Model Taxon	Angle (Deg)	Overall Length (mm)	Symphysis Length (mm)	Width (mm)
*O. tetraspis*	18.10	2.42	0.40	1.32
*C. moreletii*	19.32	2.32	0.36	1.37
*C. novaeguineae*	15.04	3.01	0.53	1.33
*C. intermedius*	15.42	3.20	0.71	1.38
*C. johnstoni*	12.78	3.69	0.83	1.30
*M. cataphractus*	16.19	3.18	0.80	1.38
*T. schlegelii*	22.15	3.68	1.94	1.42

Length, symphyseal length, angle and width for these beam models is based upon the morphology of specimens listed in **Table 2** **.1.** Note that these measurements are 1/100^th^ of the ‘volume scaled’ high resolution meshes, not actual specimen size.

The material properties of all beam models were arbitrarily set to that of structural steel (Young’s modulus of 200,000 MPa, Poisson’s ratio of 0.25 and density of 7.87 g/cm^3^). While material properties for bone are considerably different to steel, the results indicate relative performance of each beam model; additionally, under assumed linear behaviour, stresses or strains in other materials can easily be calculated from a given result. Section geometry of beams representing the rami and symphysis were circular in cross section with diameters of 0.05 mm and 0.07 mm respectively. The diameter of the symphysis was chosen so as to maintain mass, width and overall length between a model with a symphysis of zero length (i.e. where the rami meet at the anterior end of mandible) and one where symphyseal length accounts for 37.5% of overall length.

The four measurement variables explored with the beam models were all aligned in the XZ (coronal) plane - the beam models are in effect 2D models of the mandible. The third dimension is undoubtedly important in crocodilian skull biomechanics [8], [23] and is here incorporated in the hi-res FE models (see below). In the beam models, we kept dimensions in the Y (vertical) axis constant to permit the effects of variation in geometry in the XZ plane to be explored without confounding effects from variation in beam section.

### Complex Models

The third group of models were the high resolution Finite Element (hi-res FE) models generated from the CT scan data of each specimen listed in Table 1. The solid meshes of the cranium and mandible from each specimen were imported into Strand7 and form the basis for assembly of the FE models. Even though the present study focuses on mandibular biomechanics, crania were included within the model to provide accurate boundary conditions (i.e. simulations of jaw joint, muscle attachments and force vectors, bite points, etc.).

Construction of the FEMs was based upon previously published protocols [24], [25], [26], [27], [28] and are summarised here.

#### Orientation and axes

All models were orientated so the basal skull axis (which lies in the sagittal plane, and is defined rostrally by the tip of the premaxillae and caudally by the apex of the occipital condyle) was aligned with the global Z axis, and the X and Y axes aligned with the transverse and vertical axes respectively.

#### Quadrate-articular joint and gape

The mandible mesh was positioned to closely approximate the life position of the mandible relative to the cranium. The axis of rotation was defined with respect to the morphology of the quadrate condyles. On each side of the skull, the jaw joint was simulated using a beam aligned with the joint axis, connected to the articular surfaces of the quadrate and articular by rigid links (beams with infinite stiffness), and set to allow rotation around the beam’s long axis. In each model, gape was set to approximately 10 degrees (9.8 degrees +/−0.2 degrees).

#### Pterygoid buttress

In crocodilians the lateral surface of the pterygoid flanges is lined with hyaline cartilage and tightly apposes the medial surface of the mandible; in effect it acts as an ‘open joint’ and presumably buttresses against medial bending of the mandibular rami in response to the strong horizontally aligned vector components of the crocodilian jaw adductor muscles [29]. This action was simulated by a link element between the relevant surfaces on the pterygoid and mandible, which allowed all movements except medial translation of the mandible.

#### Jaw muscles

**Figure 12:**
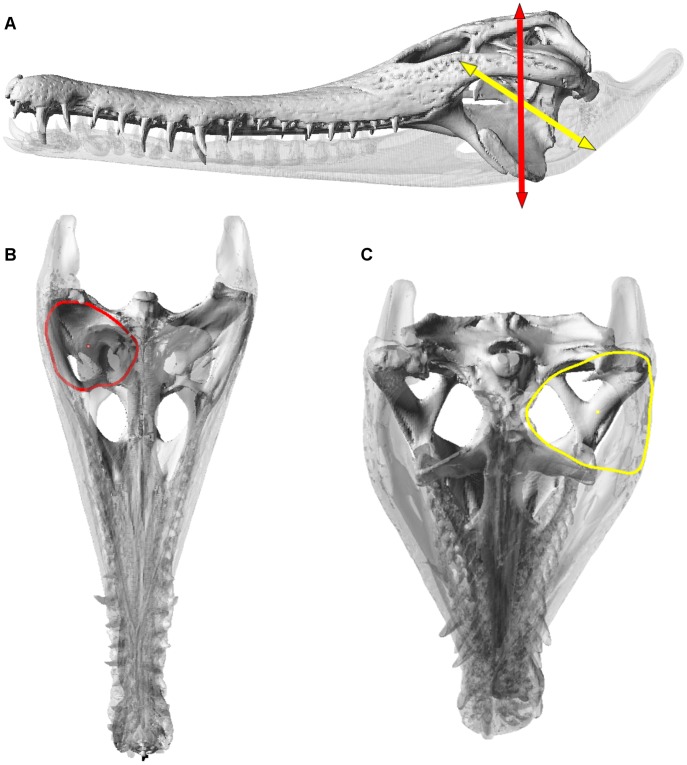
Reptile version of ‘dry skull method’ in a crocodile skull. Skull of *Mecistops cataphractus*, showing: (A), temporal (red) and pterygoid (yellow) muscle vectors; temporal vector is oriented vertically with the skull aligned horizontally, pterygoid vector runs between a point that is half of the cranial height at the postorbital bar, to the ventral surface of the mandible directly below the jaw joint. (B), calculation of the cross sectional area (CSA) for the temporal muscles; the outline maps the extent of the adductor chamber defined from osteological boundaries, viewed normal to the relevant vector. (C), calculation of CSA for pterygoid muscles; the outline is drawn normal to the vector. Outlines in B and C also show centroids, used for calculation of inlevers (see Thomason [15], McHenry [29]).

Jaw adductor musculature was simulated using truss elements that carry only tensional loads between muscle origin and insertion points [26], [27], [28]. Multiple trusses were used per muscle, with the number of elements proportional to the size of the muscle. The anatomy of muscle attachments followed descriptions in the literature [30], [31]. Muscle forces for *biting* load cases were calculated using a version of Thomason’s ‘dry skull’ method modified for crocodilian jaw muscle anatomy [15] with the ‘temporalis’ and ‘masseter’ groups [32] adjusted to ‘temporalis’ (adductor externus, adductor posterior, pseudotemporalis) and ‘pterygoid’ (pterygoidus) groups respectively [29] (Table 7). For each group, cross sectional area (CSA) was determined using osteological boundaries of the adductor chamber normal to its line of action (Figure 12), and muscle specific tension (force/area) assumed as 300 KPa [15]. The large m. pterygoidius posterior wraps around the lower jaw to insert on the retroarticular process, where its lateral extent cannot be delimited. We partially account for force from its sling-like effect on the angular by extending the ‘pterygoid’ group’s subtemporal area to the outer margin of the lower jaw (Figure 12C). Future analyses will more fully incorporate the outer part of this large muscle, which varies substantially in size between species and individuals. For now a discrete morphological proxy (lower jaw width) was judged the most precise approximation for comparing different taxa.

**Table 7:** Jaw muscle groups in crocs.

Jaw muscle	Abbreviation	Functional group	No. Beams
M. Adductor Mandibulae Externus Superficialis	MAMES	Temporal	40
M. Adductor Mandibulae Externus Medius	MAMEM	Temporal	26
M. Adductor Mandibulae Externus Profundus	MAMEP	Temporal	18
M. Adductor Mandibulae Posterior	MAMP	Temporal	46
Pseudotemporalis	PST	Temporal	8
Pterygoidus Anterior	PTA	Pterygoid	84
Pterygoidus Posterior	PTP	Pterygoid	76
Intramandibularis	IM	N/A	N/A
Depressor Mandibulae	DM	N/A	N/A

Jaw adductor muscles in crocodilians. Left column summarises the system used by lordansky (1964) [31]; left centre column shows the abbreviations for each muscle name used by Cleuren et al. (1995) [66]. Right centre, functional groupings used to generate a dry-skull method for reptiles in this study. Right, number of beams used to represent each muscle in this study.

The number of trusses used to represent each muscle group was proportional to the CSA, and within each group, the number of trusses representing each muscle were divided according to attachment area [26], [28], [29]. Muscle forces were applied as pretensions on each truss (Table 8). The diameter of each truss was calculated with respect to the measured cross sectional area of the respective muscle groups in a specimen of *C. porosus* [29]; for each specimen used here truss diameters in all models were scaled to the cube root of their volume compared to that of the *C. porosus* model. For *shaking* and *twisting* forces, we simulated an isometric force in the muscles (rather than isotonic fibre shortening during jaw adduction in *biting*) by assigning an increased elastic modulus to each truss element [29]; this had the effect of bracing the jaws as they hold a prey item, as occurs during actual shaking and *twisting* behaviours.

**Table 8:** Beam pretensions used for functional muscle groups.

	Natural size		Volume scaled	
Taxon	Temporal(N)	Pterygoid(N)	Temporal(N)	Pterygoid(N)
*O. tetraspis*	1.37	1.27	11.37	10.54
*C. moreletii*	14.04	14.04	10.40	10.40
*C. novaeguineae*	4.37	4.38	11.71	11.72
*C. intermedius*	24.70	24.65	9.81	9.79
*C. johnstoni*	1.96	1.68	11.52	9.87
*M. cataphractus*	12.34	11.53	12.34	11.53
*T. schlegelii*	10.56	12.29	11.38	13.25

Pretension values are on a ‘per beam’ basis.

#### Restraints

Free body rotation was prevented by restraining nodes on the skull - restraints prevent translation and/or rotations about a given axis. For *biting* and *shaking* restraints, a node on the apex of the occipital condyle was ‘fully fixed’ (translation and rotation) in all axes; for *twisting*, this node is fixed in translation only. For *biting*, each of the teeth involved in the bite (see below) were restrained against rotation about the jaw hinge axis (dθ); additionally the two left teeth are restrained for translation along the jaw hinge axis (dZ – i.e. laterally). For *twisting*, these teeth are all fully fixed. The surface of the occipital condyles and teeth involved in restraints were tessellated with beams to prevent point load artefacts.

#### Bite points

**Figure 13:**
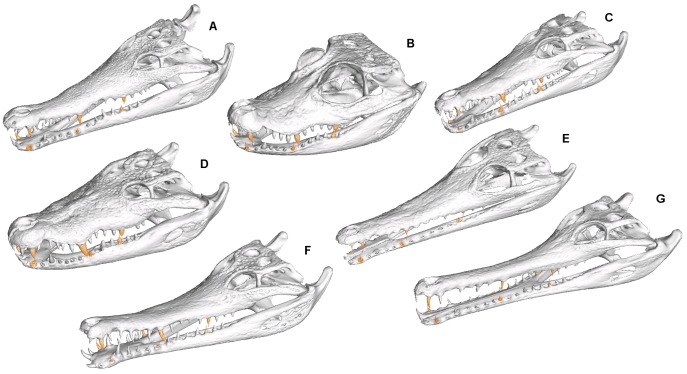
Bite points for bite, shake and twist. Teeth used in simulating front, mid and back bite points are shown in orange. *Crocodylus intermedius* (A), *Osteolaemus tetraspis* (B), *Crocodylus novaeguineae* (C), *Crocodylus moreletii* (D), *Crocodylus johnstoni* (E), *Mecistops cataphractus* (F), *Tomistoma schlegelii* (G).

For *biting, shaking* and *twisting* loads, the simulated bite point was at the front of the jaw, at the largest tooth in the premaxillary row. All four teeth (the fourth premaxillary pair from the cranium, and the fifth dentary pair from the mandible) were designated as ‘holding’ prey. For *biting* loads, ‘mid’ and ‘rear’ bites (Figure 13) were also simulated (for predictions of bite force - see below) but structural mechanical data from these is not presented here. Loads/restraints were applied to the apical node of each tooth involved in the bite point, with tessellated beams on the teeth used to reduce point load artefacts.

#### Loaded/restrained surfaces

In Finite Element modelling, single nodes to which a load or restraint are applied can be subject to very high stresses which are an artefact of the modelling technique. To reduce the effect of these ‘point load’ artefacts, the nodes on the neighbouring surface were connected by a network of beams that are slightly stiffer than the underlying bone [26], [27], [28], [29]. These networks were used at the jaw joint (to line the articular surfaces), the occipital condyle (again, lining the articular surface), on the pterygoid buttress and apposing part of the mandible, on the teeth involved with the bite point, and at the muscle attachment surfaces.

#### Material properties

The skulls were modelled with homogeneous material property sets, with the brick elements representing bone assigned an elastic (Young’s) modulus of 13,471 MPa. This value was based upon the modulus of the mean bone density in the *M. cataphractus* skull, using the conversions of Hounsfield Unit to density to modulus given by McHenry and colleagues [26]. For beam elements, the elastic modulus of the trusses representing muscle fibres was set to 0.1 MPa for *biting* load cases and 15 MPa for *shaking* and *twisting* load cases [29]. Elastic modulus of the beams used to reinforce the loaded/restrained surfaces were assigned a modulus of 100,000 MPa and a diameter of 1.92 mm in the *M. cataphractus* model, scaled accordingly in the other models. The elastic modulus of the beam representing the jaw hinge was set to a high value in order to prevent unwanted movements of the joint (Table 9).

**Table 9:** Material properties for elements used in each model.

Element	E (MPa)	ν	ρ (g/cm^3^)	Type	Ø(mm)
Pterygoid Buttress Surfaces (PBS)	100,000	0.25	0.001	Beam	1.92
Jaw Joint Surfaces (JJS)	100,000	0.25	0.001	Beam	1.92
Occipital Condyle Surface (OCS)	100,000	0.25	0.001	Beam	1.92
Jaw Hinge Axis (JHA)	200,000	0.25	7.85×10^−15^	Beam	6.39
Bite Point Surfaces (BPS)	100,000	0.25	0.001	Beam	1.92
Muscle Attachment Surfaces (MAS)	10,000	0	1.05	Beam	0.64
Intrinsic Muscle (IM)	0.1	0	1.05	Truss	6.99
Extrinsic Muscle (EM)	15	0	1.05	Truss	6.99
Bone	13,470	0.4	1.58	Brick	N/A

#### Scaling

Each model was assembled and solved at its natural size for each load case. Since the hypotheses being tested concern shape, it was necessary to control for size: this was done by rescaling each model so that the volume of cranium and mandible were the same as for the *Mecistops cataphractus* model [29], [33], which was intermediate between the smallest and largest specimens used in the analysis. In the scaled models, the diameter of all beam elements was standardised. We quantified the sensitivity of results to different scaling criteria (surface area [34] and length [35]), which are not presented here but were found to have similar strain discrepancies between specimens regardless of scaling method.

#### Load cases

*Biting* load cases were simulated by restraining teeth at the bite point and applying pretensions to the ‘muscle beams’, as described above. ‘Front’, ‘mid’, and ‘rear’ bites were simulated for unscaled (‘natural’) and scaled models; for the latter, we simulated bites where muscle forces were scaled to the 2/3 power of the change in volume (‘volume scaled’), and one where muscle forces were adjusted so that the resultant bite force was equivalent to the bite force measured from the *M. cataphractus* model (‘tooth equals tooth’, or ‘TeT’). The TeT load case thus eliminated the effects of size and load, and provides the simplest examination of the effects of shape upon skull mechanics.

**Figure 14:**
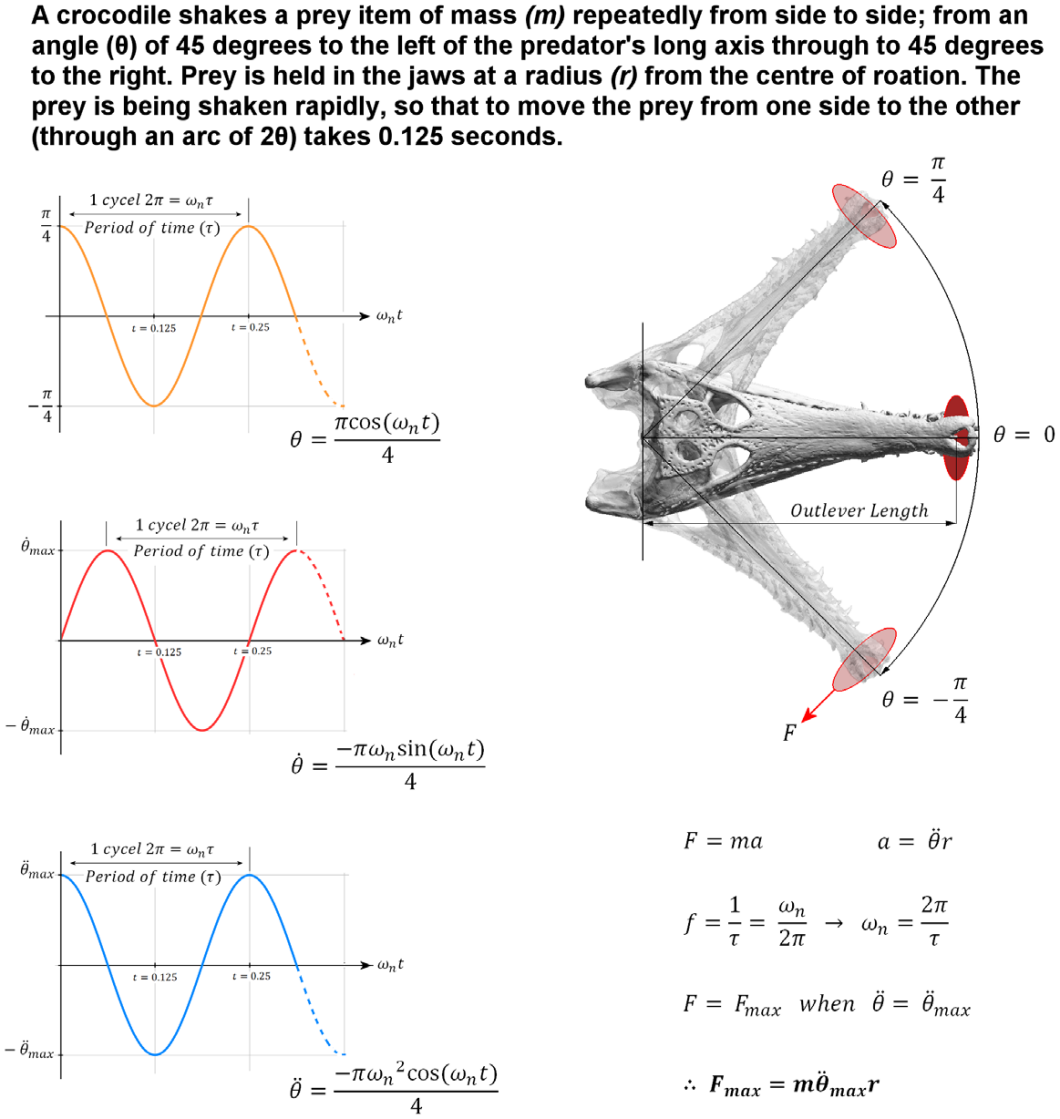
Calculation of shake forces. The problem definition used to determine the equations of motion that describe the feeding behaviour associated with *shaking* a prey item. This motion is considered to be harmonic; since the skull oscillates about a neutral axis in a set period of time (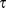); in our case this period is 0.25 seconds – i.e at a frequency (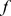) of 4 full cycles per second. Left: the equations of motion associated with *shaking*, where 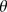 is angular displacement, 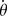 is angular velocity and is angular acceleration. Maximum angular acceleration (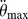) occurs each time the skull changes direction; in our case (radians/sec^2^), where a positive value indicates counter clockwise acceleration and a negative value indicates a clockwise acceleration. Right: the range of motion for a crocodile *shaking* a prey item. Bottom right: shows the equations used to calculate the maximum force (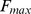) exerted on the skull as a result of *shaking* a prey item of mass (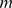) – approximately 2.55 kg in the *M. cataphractus* example shown here. Here 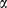 denotes linear acceleration (in the direction of force 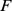) and 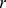 denotes the distance to the centre of rotation. For our calculations 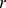 is calculated as the perpendicular distance from the jaw hinge axis to the centre of mass of the prey item (outlever length) – approx. 297 mm in *M. cataphractus*.

For *shaking* load cases, a lateral force was applied to each of the teeth at the bite point; the magnitude of the force was initially calculated for each model on the basis of prey approximately three times the mass of the skull being held at the front of the jaws, and shaken from side to side at a frequency of 4 full cycles per second ([29]; Figure 14). The force magnitudes calculated for the *M. cataphractus* model were then applied to the volume rescaled models.

**Figure 15:**
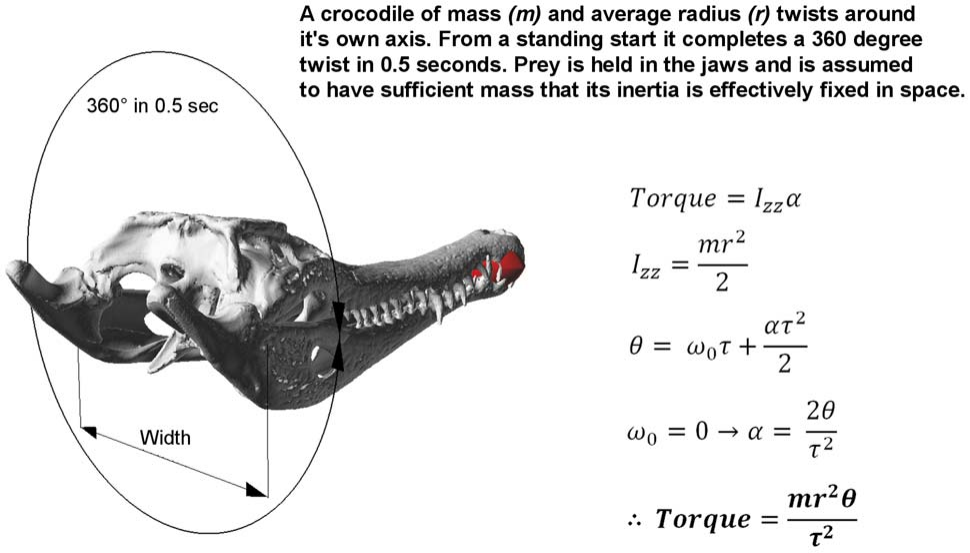
Calculation of twist forces. The problem definition used to determine the equations of motion that describe the feeding behaviour associated with *twisting* a prey item. Bottom Left: the range of motion for a crocodile *twisting* a prey item. Bottom right: the equations used to calculate the Torque generated by a crocodile of mass (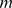) as a result of *twisting* about its own axis with a prey item held in its jaws. Torque is the produce of moment of inertia (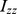) about the animals long axis and the angular acceleration (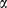) – which is assumed to be constant. Moment of inertial is calculated using mass (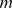) and radius (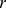); in our calculations mass is approximated as fifty times the mass of the skull (approx. 40 kg in the *M. cataphractus* example shown here), while radius is approximated as skull width (approx. 152 mm in *M. cataphractus*). Initial angular velocity (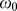) is zero since in this case the twist is being made from a standing start. 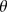 denotes the angular displacement of the twist in radians (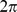 or 360 degrees in this case), while 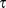 denotes the time taken to complete the rotation –0.5 seconds.

Similarly, the *twisting* load case was calculated on the basis of a large prey item being held in the jaws, with the crocodilian imposing a torsion load on the bite point by rotating its postcranium about its own long axis at a rate of 2 full rotations per second (Figure 15). This torque was then simulated by fully fixing the teeth at the bite point and applying the calculated moment to the occipital condyle. The moment calculated for the *M. cataphractus* model was applied to the volume rescaled models.

### Assessing Biomechanical Performance

**Figure 16:**
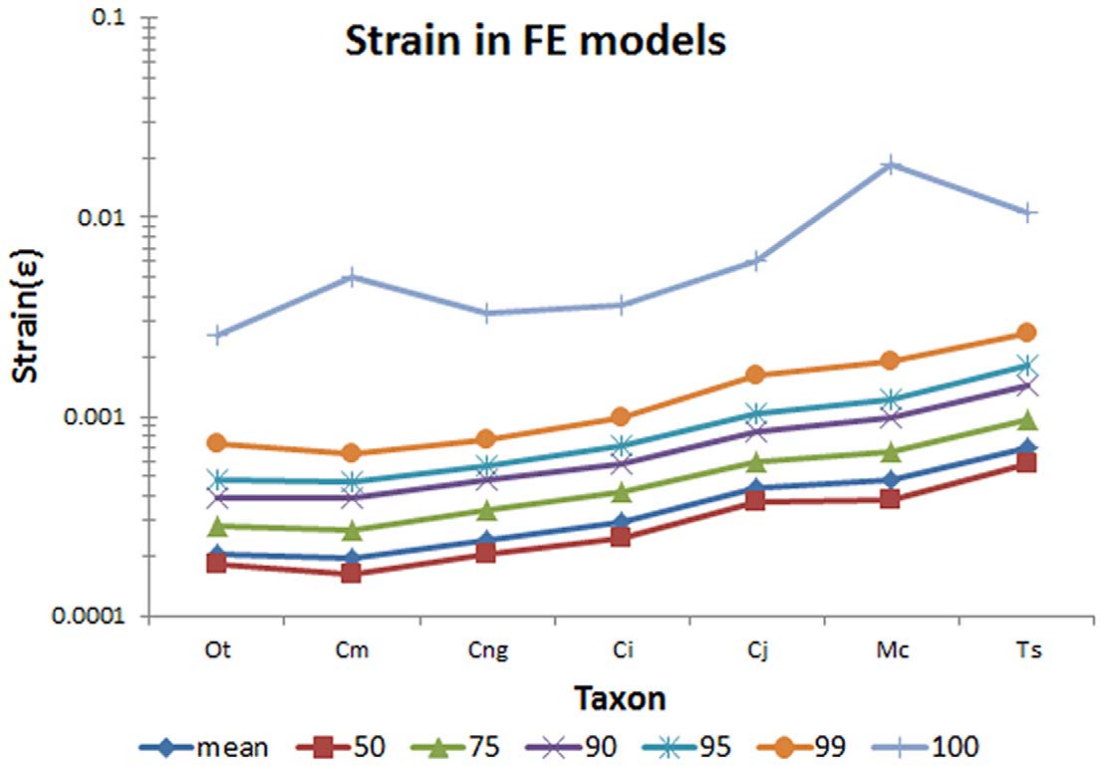
Values of strain from complex FE models. Shows mean, 50%, 75%, 90%, 95%, 99% and 100% strain values for taxon used in this study. 95% strain represents the largest elemental value of strain in the model if the highest 5% of all values are ignored. 100% strain is the maximum elemental strain in the model and likely represents constraint artefacts caused by boundary conditions. Taxon abbreviations: Ot, *Osteolaemus tetraspis*; Cm, *Crocodylus moreletii*; Cng, *Crocodylus novaeguineae*; Ci, *Crocodylus intermedius*; Cj, *Crocodylus johnstoni*; Mc, *Mecistops cataphractus*; Ts, *Tomistoma schlegelii*.

For each load case in each complex FE model, strain values for the tetrahedral brick elements making up the skull and cranium were exported as text files and analysed in the R statistical programming environment [36]. Since we wished to determine the strength of the mandibles under load, the maximal strain values are the most useful for statistical analyses. In complex FE models, however, the maximal strain values are often associated with artefacts of the model (e.g. restraints, load points, and elements with high aspect ratio geometry). We therefore used the 95% values of strain in each model [37] which provide a similar pattern of results as the mean, median, 75%, and 90% values but differ from the 100% (i.e. maximal) values (Figure 16); in the absence of validated data on actual strain values our assumption that 95% values provide a suitable basis for the analysis of results is untested but is logically sound. Contour plots of von Mises strain were also used to provide a visual comparison of results.

The beam models each comprised three elements and are not subject to the artefacts seen in the complex FE models. Results were collected as maximal fibre stress and converted to strain using elastic modulus and the equation 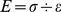, where 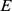 represents elastic (Young’s) modulus, 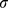 represents stress, and 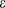 represents strain.

Bite force was measured as the sum of the absolute values for nodal reaction forces for the four bite points involved in each bite, measured as reaction force in the rotational direction of the jaw hinge axis (‘Dθ’ in Strand7).

### Statistical Evaluation of Models

Analysis focused upon quantifying correlations between morphometric data and strain values, using natural logarithms of linear measurements, strain data, and principal component (PC) values. Scatter plots of strain vs morphometric variables were produced using Excel (v2010, Microsoft).

Size corrected (by centroid) landmark data was analysed using principal components analysis (PCA). Visualisation of shape change along PC axes was performed using Morphologika v2.5, [21]. The eigenscores from PCA represent relative shape variation and are used here as descriptors of shape as defined by Kendall [38], and is all that remains after rotation, translation and scale are removed; see [38], [39], [40]. Only the first two principal components were used in this analysis because the first two PC values accounted for 92% of shape variation (66% PC1, 26% PC2) and low sample size limits the number of explanatory (morphometric) variables that can be evaluated.

For each type of mandible load (*biting*, *shaking*, or *twisting*), we evaluated the explanatory power of linear measurements compared with shape. Each linear measurement was tested as an explanatory model (EM) and compared using the second-order Akaike’s Information Criterion, AICc, as recommended in the case of small sample sizes [41], [42], [43]. AICc score is a measure of the relative amount of information lost when using an explanatory model to approximate reality, taking into account both the number of parameters in the EM and the sample size. A lower AICc score indicates a better EM, however interpretation is not entirely clear cut and there can be some uncertainty as to how much “better” one EM is than another and instead a few EM can be considered AICc-best. We have reported the estimated parameters of each EM, the log-likelihood of each EM, AICc, ΔAICc, and the Akaike weights. ΔAICc values are the difference in AICc between an explanatory model and the AICc-best explanatory model. EMs within 2 of each other were considered nearly identical in information, while EMs with ΔAICc values of 4 and 8 are considered fair and EMs with a ΔAICc greater than 10 are poor [44]. Akaike weights can be interpreted as approximations of the EM selection probabilities or posterior probabilities of the EM [44]. Effectively, Akaike weights are a measure of the relative informativeness of each EM. Analysis was performed within the R statistical programming environment version 2.15.0 [36] using the ‘shapes’ [45] and ‘MuMIn’ [46] packages.

Linear morphometric variables were selected *a priori* on the basis of beam theory principles. For *biting*, we evaluated mandibular length, the eigenscores of the first principal component, and the eigenscores of the first two principal components. For *shaking* and *twisting*, we evaluated mandibular length, symphyseal length, mandibular angle, and eigenscores of the first two principal components.

## Results

### Shape Analysis

**Figure 17:**
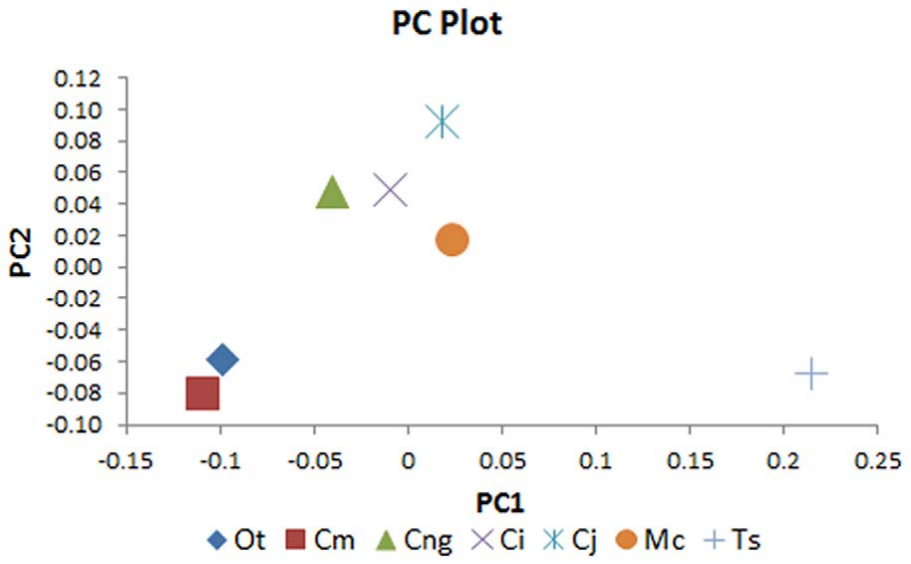
Principal component plot. Principal component 1 (PC1) versus principal component 2 (PC2) from geometric morphometric analysis Taxon abbreviations: Ot, *Osteolaemus tetraspis*; Cm, *Crocodylus moreletii*; Cng, *Crocodylus novaeguineae*; Ci, *Crocodylus intermedius*; Cj, *Crocodylus johnstoni*; Mc, *Mecistops cataphractus*; Ts, *Tomistoma schlegelii*.

Measurements of morphological variables are shown in Table 10. Figure 17 shows the plot of PC1 vs PC2 scores for the seven specimens. Most of the specimens lie within a defined linear band of PC values, with the exception of *Tomistoma schlegelii* which appears to be an outlier (Figure 17). *T. schlegelii* is clearly separated from the other specimens along the PC1 axis, but not on the PC2.

**Table 10:** Length, Symphyseal Length, Angle and Width for each of the mandibles used in this study.

Taxon	Angle (Deg)	Overall Length(mm)	Jaw Hinge Length(mm)	Symphysis Length(mm)	Width(mm)
*O. tetraspis*	13.91	266.21	234.05	35.15	148.75
*C. moreletii*	13.75	273.49	229.43	35.72	158.30
*C. novaeguineae*	10.47	346.68	298.81	52.84	143.48
*C. intermedius*	10.07	363.58	314.93	66.60	151.23
*C. johnstoni*	9.71	411.38	364.25	80.09	147.05
*M. cataphractus*	11.72	369.40	309.88	77.70	152.26
*T. schlegelii*	18.37	413.61	362.93	191.96	160.90

Linear measurements displayed above for each specimen when scaled to the same volume as *M. cataphractus.*

**Figure 18:**
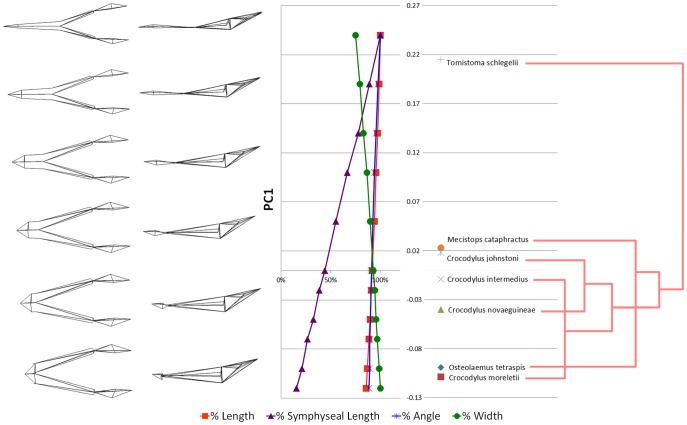
Quantification of Principal Component 1 (PC1). Wireframe (left) of mandible from dorsal and lateral perspectives illustrates the change in shape along PC1 axis. Note the longer symphyses at higher PC1 values. The chart in the centre shows the value of each morphological variable (e.g. symphyseal length) at a given PC value, as a percentage of the maximal value for that morphological variable. Specimens are plotted according to their respective PC1 values (centre right). Phylogram (right) shows poor correlation of specimen PC1 scores with phylogeny. Phylogeny based upon the results of Erickson and colleagues [47].

**Figure 19:**
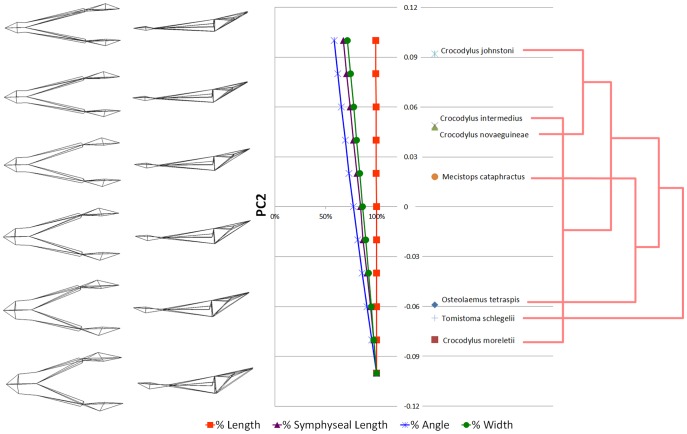
Quantification of Principal Component 2 (PC2). Wireframe (left) of mandible from dorsal and lateral perspectives illustrates decreasing mandible robustness with increasing PC2 values. The chart in the centre shows the value of each morphological variable (e.g. symphyseal length) at a given PC value, as a percentage of the maximal value for that morphological variable. Specimens are plotted according to their respective PC2 values (centre right). Phylogram (right) shows poor correlation of specimen PC2 scores with phylogeny. Phylogeny based upon the results of Erickson and colleagues [47].

The morphological components of the principal components are shown in Figures 18 and 19. Symphyseal length (SL) shows the greatest percentage change along the PC1 axis, with some change in width (W) but only minor changes in angle (A) and length (L) (Figure 18). Along PC2, angle shows the highest percentage change, but SL and W are nearly as great (Figure 19). Width is inversely correlated with the other variables along PC1, whereas along PC2 changes in SL, W, and A are correlated. Length does not change along PC2. Correlation with phylogeny is poor along both PC axes (Figures 18 and 19), suggesting that symphyseal length is not strongly constrained by phylogeny, although we did not test this statistically (due to small sample size).

### Bite Force

**Figure 20:**
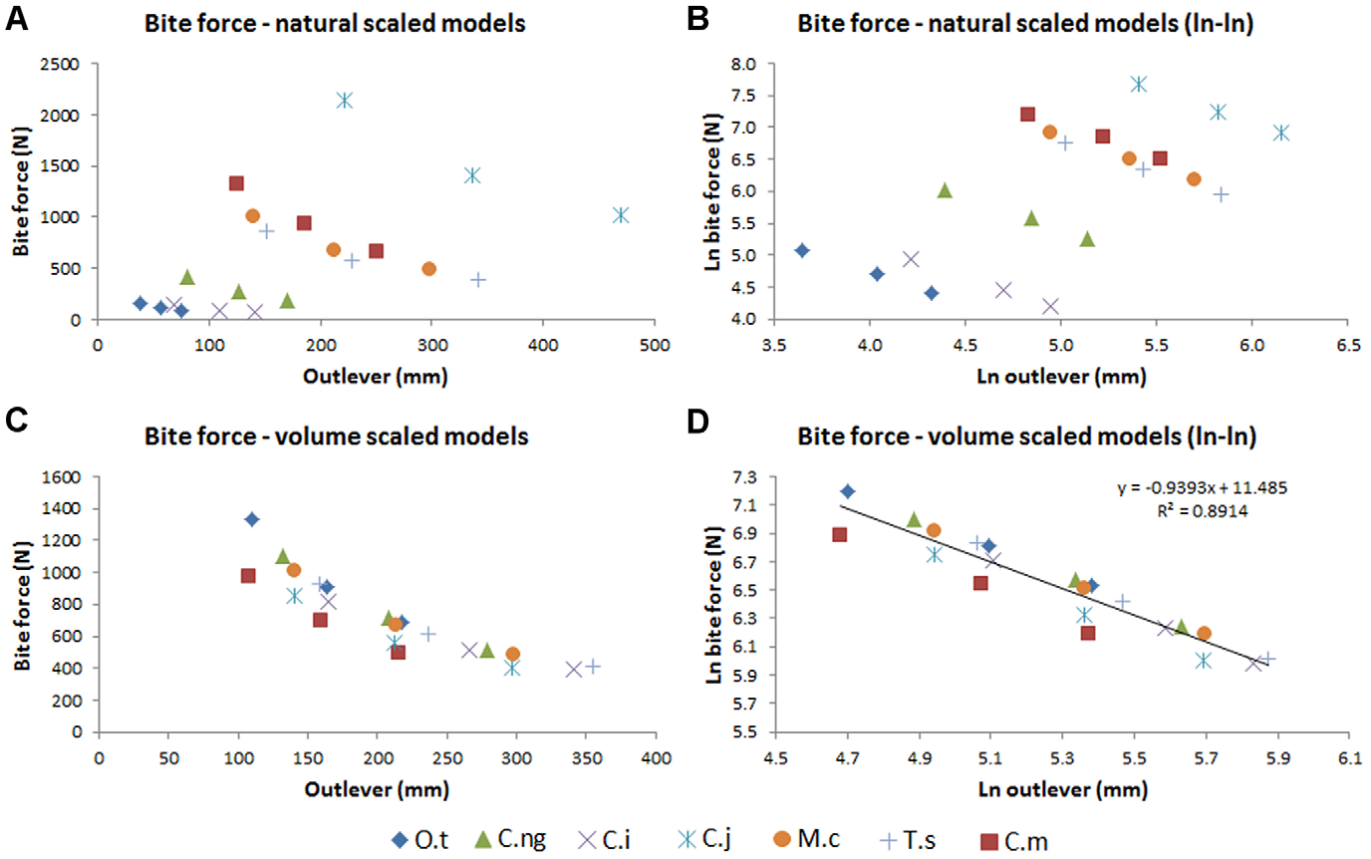
Bite force estimates for high resolutions FEMs. Estimates of bite force generated by the high resolution FEMs, plotted against outlever length (distance from jaw hinge axis to bite point). Charts to right show natural logarithm transformed data. (A) and (B) show results from models at ‘natural’ sizes, (C) and (D) show results from models rescaled to the volume of the *M. cataphractus* model. Note the strong correlation between volume-scaled bite force and outlever (D). Front, mid, and rear bites for each FEM are shown. Taxon abbreviations: O.t, *Osteolaemus tetraspis*; C.ng, *Crocodylus novaeguineae*; C.i, *Crocodylus intermedius*; C.j, *Crocodylus johnstoni*; M.c, *Mecistops cataphractus*; T.s, *Tomistoma schlegelii*; C.m, *Crocodylus moreletii*.

Bite force predictions from the hi-res FEMs are given in Table 11 and plotted in Figure 20. The maximum estimated bite force, 2145 N for a rear bite by the *C. intermedius* ‘natural’ sized model is considerably less than that reported for that taxon (6276 N for an animal by Erickson [47]). Scaled to skull volume, the relationship between bite force and outlever length appears to be consistent between taxa, with the results for most specimens falling close to the regression line for the logarithm transformed data, suggesting that bite force is related to head size and bite point. The slight non-linearity (slope of the regression line of logarithm transformed data is −0.93) in the data is not expected from the basic lever mechanics that are sometimes used to model bite force [15], [48] and may stem from the measurement of bite force in the rotational axis of the jaw hinge; any component of the joint reaction force not aligned with that axis will be ignored by this measurement.

**Table 11:** Bite force estimates for natural sized and volume rescaled models.

	Natural Size (N)			Volume Scaled (N)		
Taxon	Front	Mid	Back	Front	Mid	Back
*O. tetraspis*	82.53	109.31	160.61	685.35	907.57	1333.49
*C. moreletii*	660.82	935.99	1317.57	489.93	693.04	976.52
*C. novaeguineae*	191.85	266.77	410.96	513.52	714.07	1099.80
*C. intermedius*	1015.22	1403.02	2144.98	403.42	557.24	851.82
*C. johnstoni*	67.06	86.59	139.25	395.99	510.51	820.36
*M. cataphractus*	485.86	672.55	1009.16	485.92	672.61	1009.25
*T. schlegelii*	380.31	568.86	860.26	410.42	613.28	927.27

### Simple Beam Models #1

**Figure 21:**
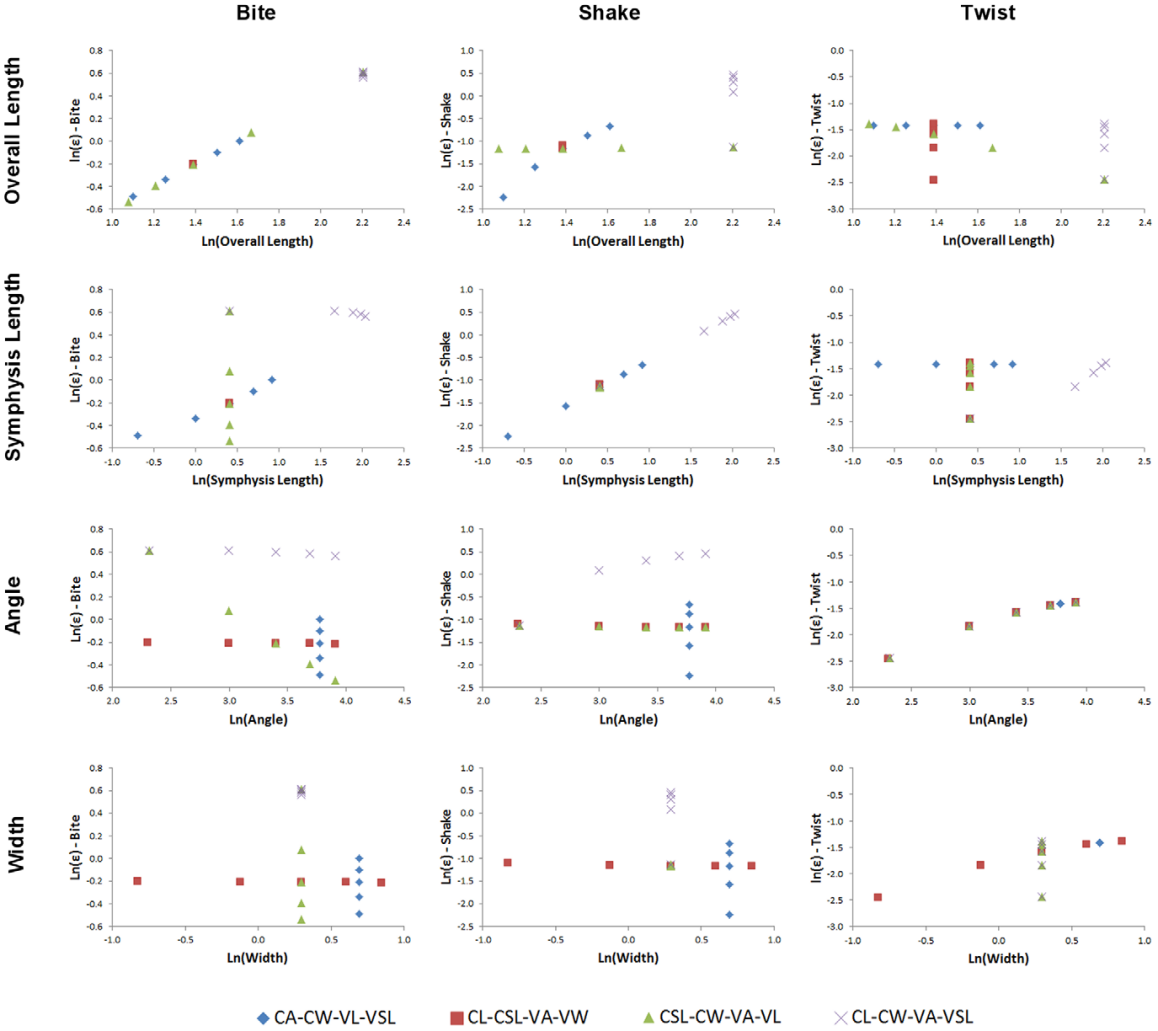
Strain for simple beam models #1. Strain in the first set of simple beam models, plotted against morphological variables (from top) length, symphyseal length, angle, and width, for *biting* (left), *shaking* (middle) and *twisting* (right) loads. Note the strong correlation between bite and overall length, shake and symphyseal length, and twist and angle. Data is plotted as natural logarithms of linear measurements (mm) and angles (degrees). Model abbreviations are as follows: (CL-CSL-VA-VW) – Constant length and symphyseal length, variable angle and width. (CL-CW-VSL-VA) – Constant length and width, variable symphyseal length and angle. (CA-CW-VSL-VL) – Constant angle and width, variable symphyseal length and length. (CSL-CW-VL-VA) – Constant symphyseal length and width, variable length and angle.

Results for the first set of beam models are shown as charts of strain values plotted against the value of each morphological variable (L, SL, A, W) in turn, for *biting*, *shaking*, and *twisting* (Figure 21).

Under simulated bite loads, strain in the beam models correlated positively and linearly with length when symphyseal length also varied (CA-CW-VL-VSL), and with length when symphyseal length did not vary (CSL-CW-VA-VL). There was a strong non-linear negative correlation of strain with angle when length also varied (CSL-CW-VA-VL), and a weak non-linear negative correlation with angle and symphyseal length when these co-varied (CL-CW-VA-VSL). There was no correlation with width. The factors determining strain in the beam models under *biting* are thus mainly length, with the covariance of angle and symphyseal length showing a weak effect when length is held constant.

Under shake loads, strain correlated positively (although non-linearly) with length when symphyseal length also varied (CA-CW-VL-VSL), but did not correlate with length when symphyseal length was held constant (CSL-CW-VA-VL). Correlation with symphyseal length was positive and linear for models where symphyseal length varied (CA-CW-VL-VSL), but strain did not vary between models when symphyseal length was constant (CL-CSL-VA-VW, CSL-CW-VA-VL). Correlation with angle was positive and non-linear only when symphyseal length covaried (CL-CW-VA-VSL). There was no correlation with width. The factor determining strain in the beam models under *shaking* is thus principally symphyseal length.

Under twist loads, strain correlated negatively and non-linearly with length when angle and length varied (CSL-CW-VA-VL), positively and linearly with symphyseal length when symphyseal length and angle covaried (CL-CW-VA-VSL), positively and non-linearly with angle when angle varied (CL-CSL-VA-VW, CSL-CW-VA-VL, CL-CW-VA-VSL), and with width when angle covaried (CL-CSL-VA-VW). Strain did not correlate with length or symphyseal length when angle did not vary. The factor determining strain values in the beam models under *twisting* appears therefore to be angle.

### Beam Models #2

**Figure 22:**
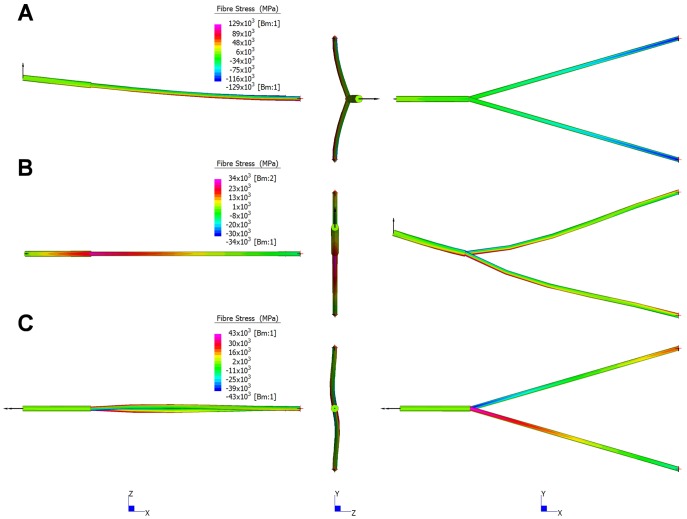
Stress contour plots for beam models. Stress contour plots for beam model based on *M. cataphractus* for *biting* (A), *shaking* (B), and *twisting* (C) loading regimes. The models are shown from lateral (left), anterior (middle) and dorsal (right) views. The regions of high tensile (reds) and compressive (blues) stresses are shown. Deformations are exaggerated to better illustrate the structural response to loads. The general pattern of strain is similar for all beam models.

Contour plots illustrating regions of high tensile (positive) and compressive (negative) fibre stress for the *M. cataphractus* beam model under *biting*, *shaking*, and *twisting* loads are shown in Figure 22. Deformations are exaggerated to emphasize the structural response to each load simulated and the general pattern of stress is characteristic of all beam models. Under *biting*, the mandible experiences highest stress posteriorly on the rami, which decreases anteriorly along the mandible (Figure 22A). For *shaking*, the highest stress is located at the symphyseal-rami junction, decreasing in both the anterior and posterior directions (Figure 22B). For *twisting*, stress in the symphysis is uniform along its length, with highest stress in the anterior portion of the rami (peaking at the symphyseal-rami junction), where it decreases before increasing, posteriorly along the rami (Figure 22C).

**Figure 23:**
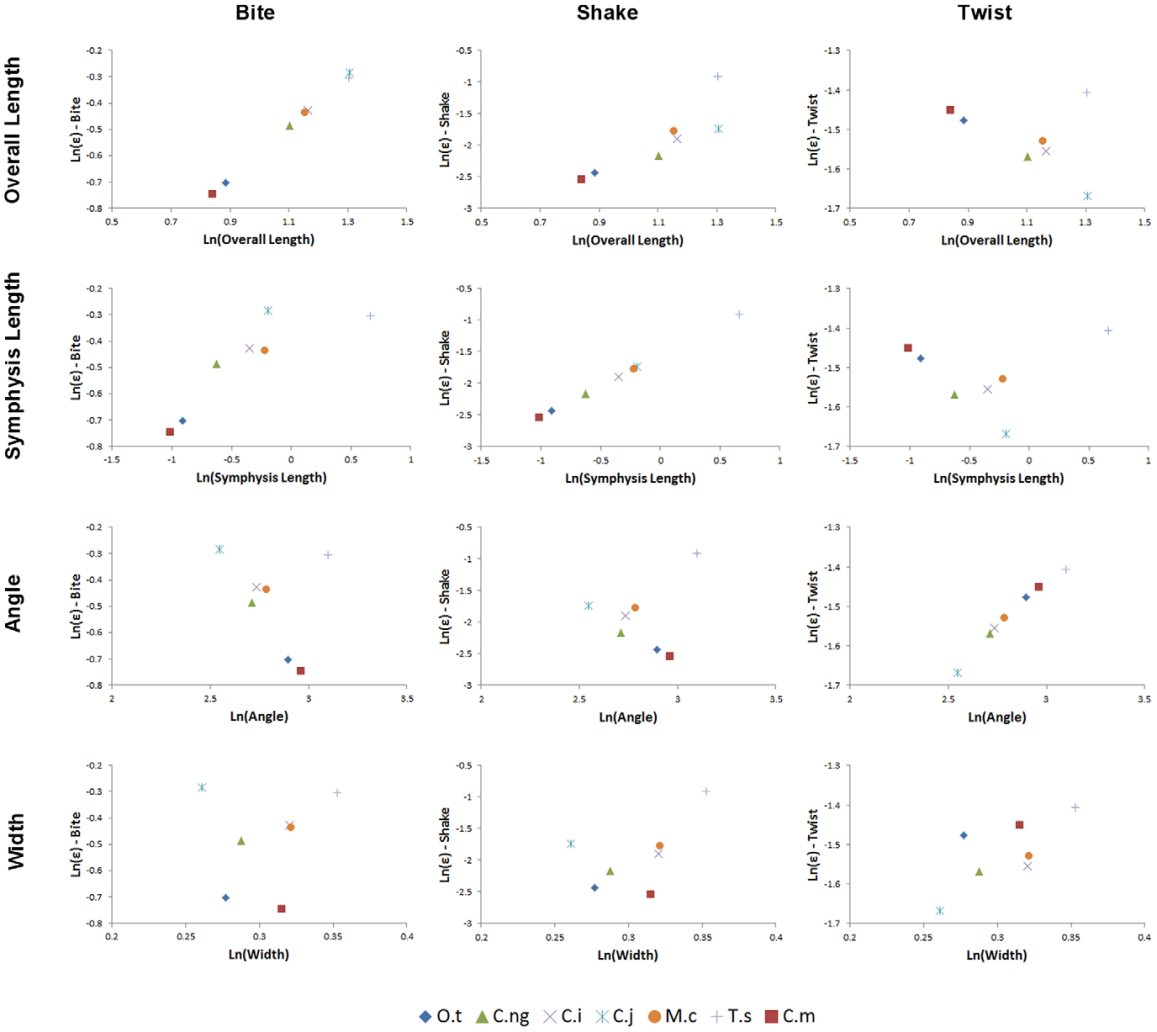
Strain for simple beam models #2. Strain in the second set of simple beam models, plotted against morphological variables (from top) length, symphyseal length, angle, and width, for *biting* (left), *shaking* (middle) and *twisting* (right) loads. Note the strong correlation between bite and overall length, shake and symphyseal length, and twist and angle. Data is plotted as natural logarithms of linear measurements (mm) and angles (degrees). Dimensions of the beam models are based upon the volume rescaled versions of the high resolution FEMs for the corresponding species. Taxon abbreviations: O.t, *Osteolaemus tetraspis*; C.ng, *Crocodylus novaeguineae*; C.i, *Crocodylus intermedius*; C.j, *Crocodylus johnstoni*; M.c, *Mecistops cataphractus*; T.s, *Tomistoma schlegelii*; C.m, *Crocodylus moreletii*.

Maximum strain for the second set of beam models is shown in Figure 23, plotted against the morphological variables, for *biting*, *shaking* and *twisting*, as log transformed data. The plots show a clear correlation between; length and *biting*, symphyseal length and *shaking*, and angle and *twisting*. AICc values confirm that, for *shaking* and *twisting* respectively, symphyseal length and angle are by far the best explanatory variables, with very low AICc values and Akaike weights close to 1.0 (Tables 12 and 13). Weaker correlations are apparent between symphyseal length and *biting*, as well as between length and *shaking*, although the latter is a relatively poor explanatory model based on AICc. In the plots of strain against length and symphyseal length in *twisting*, *T. schlegelii* appears to be an outlier while the data points for the other specimens suggest a negative relationship between length and symphyseal length for strain in *twisting*, but again these lack explanatory power under Akaike scores.

**Table 12:** Comparison of morphological variables for predicting shake strain in a simplified beam representation of a crocodile mandible.

	Intercept	Ln(Length)	Ln(Symphyseal L.)	Ln(Angle)	df	logLik	AICc	ΔAICc	Akaike weight
**2**	−1.55		0.97		3	32.12	−50.24	0.00	1.00
**1**	−4.78	2.58			3	−0.21	14.43	64.67	0.00
**3**	−3.67			0.62	3	−5.01	24.02	74.26	0.00

Morphological variables are in order with AICc-best first. Columns correspond to parameter estimates for each model, log-likelihood of model given data, AICc scores, ΔAICc from AICc-best, and Akaike weight.

**Table 13:** Comparison of morphological variables for predicting twist strain in a simplified beam representation of a crocodile mandible.

	Intercept	ln(Length)	ln(Symphyseal L.)	Ln(Angle)	df	logLik	AICc	ΔAICc	Akaike weight
**3**	−2.86			0.47	3	21.57	−29.13	0.00	1.00
**1**	−1.32	−0.18			3	8.29	−2.58	26.55	0.00
**2**	−1.52		0.02		3	7.75	−1.51	27.63	0.00

Morphological variables are in order with AICc-best first. Columns correspond to parameter estimates for each morphological variable, log-likelihood of morphological variable given data, AICc scores, ΔAICc from AICc-best, and Akaike weight.

### Complex FE Models

**Figure 24:**
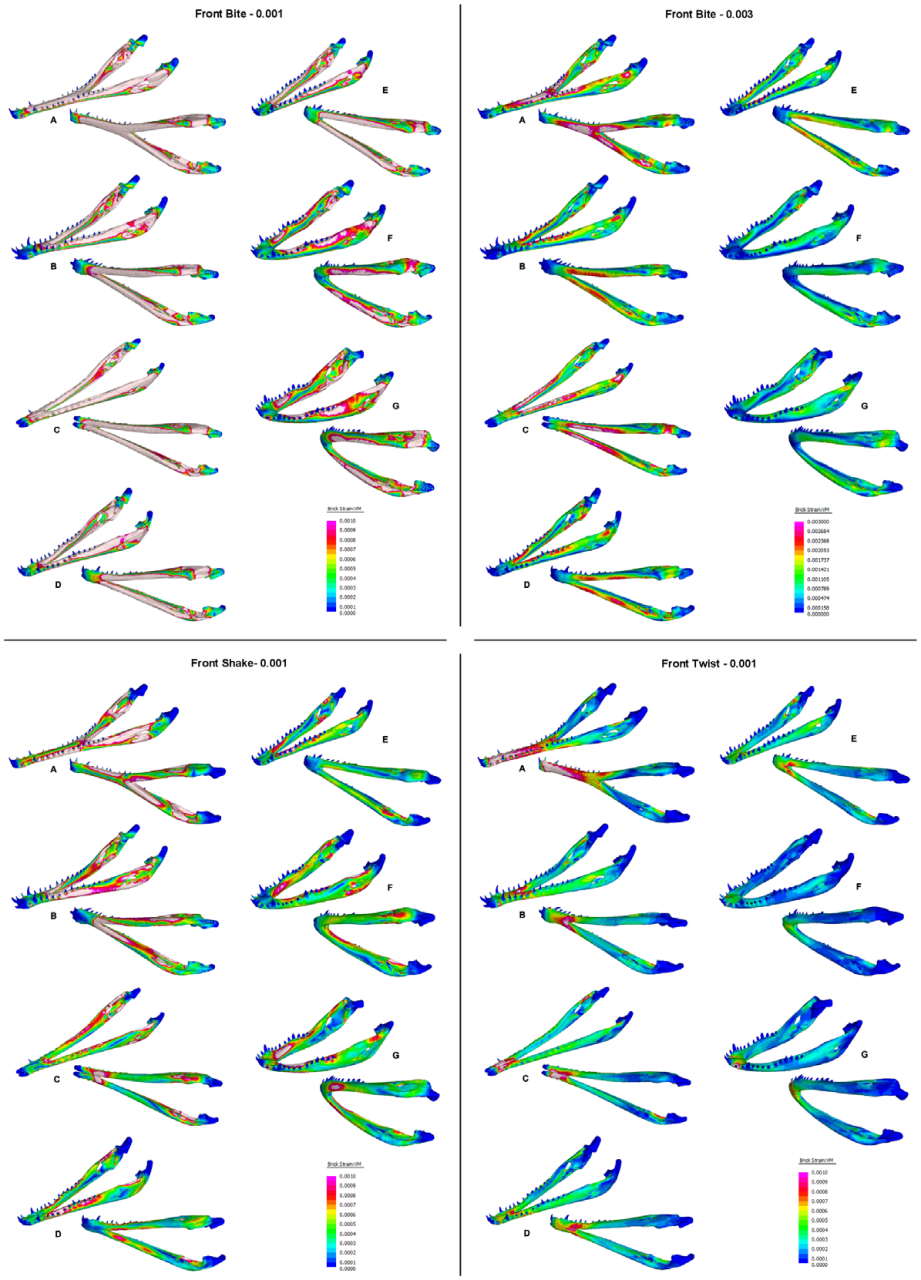
Strain plots for volume scaled FEMs. Strain plots for volume scaled FEMs under *biting*, *shaking*, and *twisting* loads to show details of strain patterns. Top: *biting* load case plotted with a maximum strain limit of 0.001 (left) and 0.003 (right); the latter limit shows the position of the peak strains, and the former gives best comparison between the different load cases. Bottom left: *shaking* load case plotted with a maximum strain limit of 0.001. Bottom right: *twisting* load case plotted with a maximum strain limit of 0.001. Taxa: A, *Tomistoma schlegelii*; B, *Mecistops cataphractus*; C, *Crocodylus johnstoni*; D, *Crocodylus intermedius*; E, *Crocodylus novaeguineae*; F, *Crocodylus moreletii*; G, *Osteolaemus tetraspis*.

Figure 24 shows strain plots for the complex FEMs under *biting*, *shaking*, and *twisting* loads. In *biting* (TeT) loads, strain is higher in longirostrine mandibles and is highest in *Tomistoma*. For all mandibles except *Tomistoma*, strains are highest in the rami, with peak strain in the anterior regions of the rami (near the symphysis), and anterior to the joint surface of the articular. Strain in the symphysis of these models is low, but strain in the rami immediately posterior to the symphysis is high, and the symphyseal-rami junction appears to be a weak point. In *Tomistoma*, strain is high in the rami, similar to the other models, but strain is also high in the symphysis; the strain pattern in *Tomistoma* is qualitatively different to the pattern in the other taxa. All of the mandibles seem to be behaving as beams, with high strains on the upper and lower edges of the mandibles and a simple neutral surface of low strain running along the length of the mandible between these edges.

For *shaking* loads, strain is high in the anterior part of the mandible but the peak strain is more concentrated at the symphyseal-rami junction than in *biting*, and unlike the situation in biting the posterior part of the symphysis is included in the region of highest strain. In the *Tomistoma* mandible strain is also high along each side of the symphysis, unlike the other models.

For *twisting* (TeT) loads, strain is highest at the symphyseal-rami junction, again with the exception of the *Tomistoma* model where the highest strains are at the anterior end of the symphysis. In all models, strain is low along the rami, and is concentrated within the symphysis. In *twisting* strain magnitude for *Tomistoma* is much higher than other specimen and the pattern of strain contours is qualitatively different.

**Figure 25:**
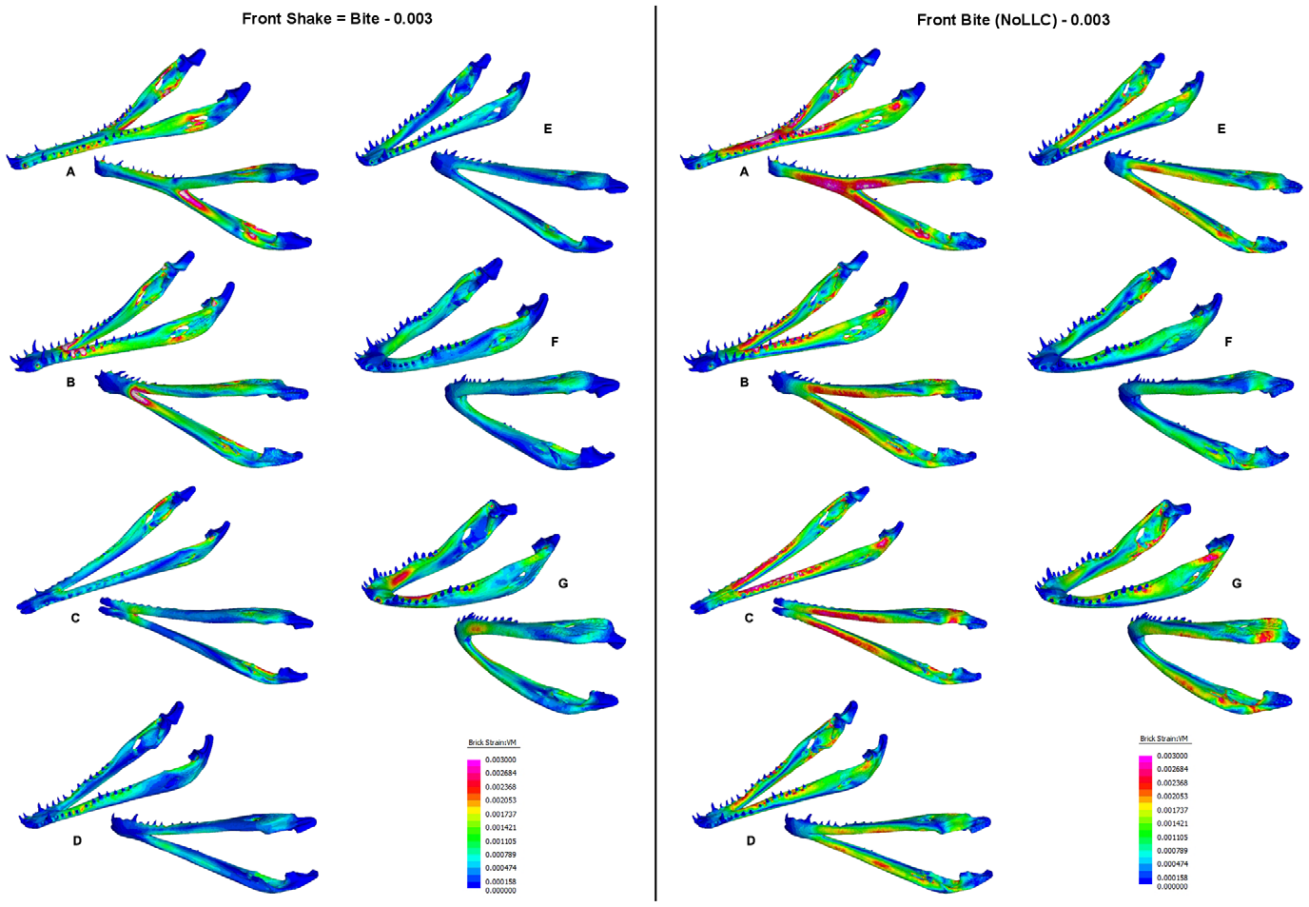
Strain plot response to equal *biting* and *twisting* loads. Direct comparison of mandible response to equal *biting* and *shaking* loads at the most anterior bite point (front). Strain magnitude is higher under the *biting* loads; the difference is noticeable for longirostrine (A–C) and mesorostrine (D–F) taxa. Taxon labels: A, *Tomistoma schlegelii*; B, *Mecistops cataphractus*; C, *Crocodylus johnstoni*; D, *Crocodylus intermedius*; E, *Crocodylus novaeguineae*; F, *Crocodylus moreletii*; G, *Osteolaemus tetraspis*.

When shake force is adjusted to match bite force (Figure 25), mandibular strain is higher under *biting* than under *shaking*, for all species. The difference is marked for most of the models, with strain in *biting* and *shaking* being most similar for the *C. moreletii* mandible.

**Figure 26:**
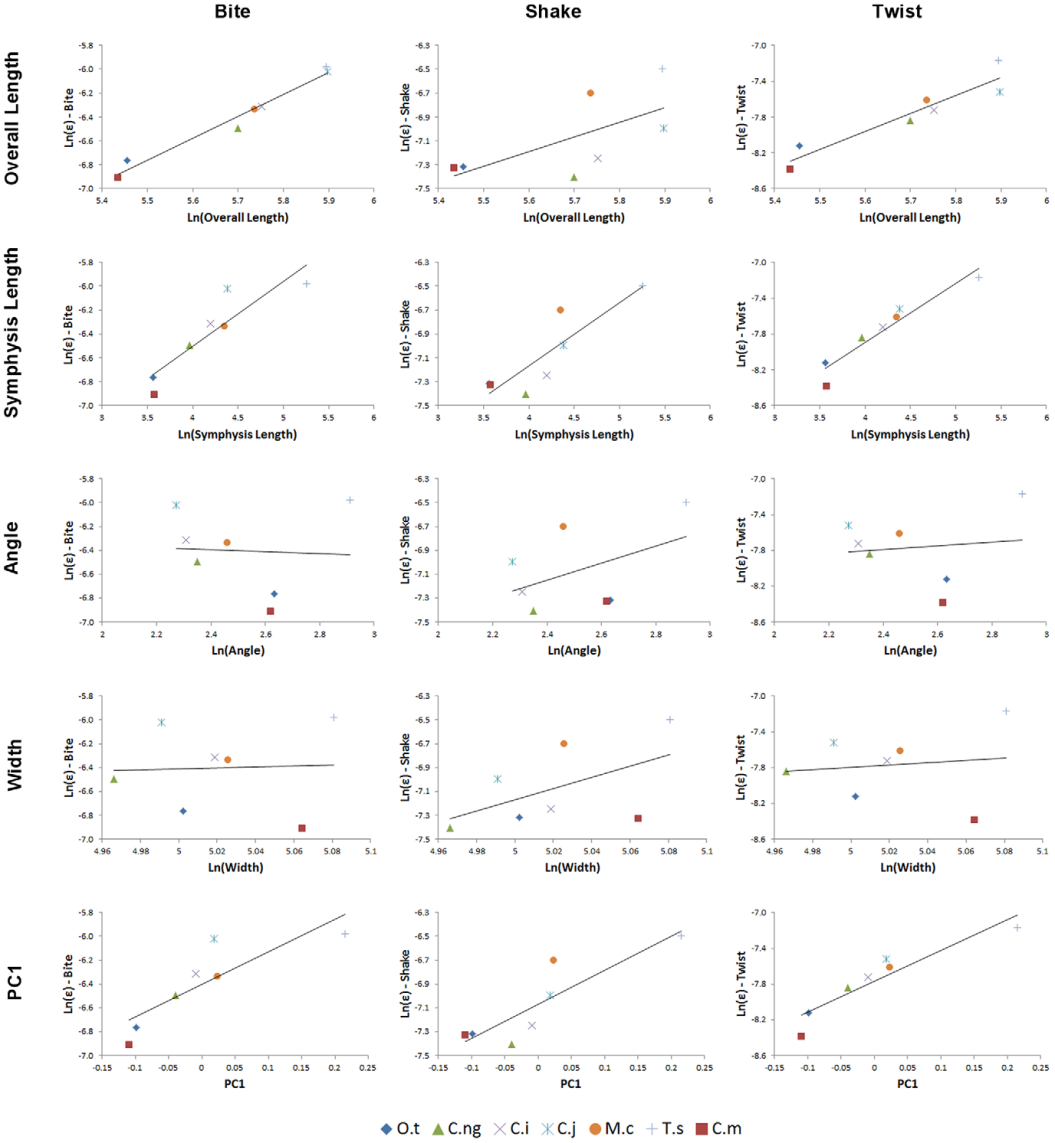
Peak mandibular strain (95% values). Peak mandibular strain (95% values) plotted against morphometric variables (from top) length, symphyseal length, angle, width, and PC1 score for *biting* (left), *shaking* (middle) and *twisting* (right) loads. Note that strain in *biting* correlates strongly with overall length and very poorly with both angle and width, whilst in *shaking* strain has reasonable correlations with both symphyseal length and PC1. Contrary to beam predictions strain in *twisting* correlated strongly with symphyseal length and very poorly with angle. Data is plotted as natural logarithms of linear measurements (mm) and angles (degrees). Taxon: O.t, *Osteolaemus tetraspis*; C.ng, *Crocodylus novaeguineae*; C.i, *Crocodylus intermedius*; C.j, *Crocodylus johnstoni*; M.c, *Mecistops cataphractus*; T.s, *Tomistoma schlegelii*; C.m, *Crocodylus moreletii*.

Peak strain (95%) values are plotted against morphometric variables in Figure 26. Under *biting*, charts suggest that strain correlates strongly with Length, and also with PC1 and symphyseal length. In *shaking*, strain correlates with symphyseal length and PC1, whilst in *twisting* strain correlates with symphyseal length, length, and PC1.

AICc scores are shown in Tables 14, 15, 16. The AICc-best explanatory model (EM) of strain in *biting* is that with length as the sole predictor (Table 14). The other two predictors, using the eigenscores from geometric morphometric analysis both have large ΔAICc values (greater than 10) and thus cannot be interpreted as effective predictors of bite strain. Note that symphyseal length was not assessed as a predictor for *biting* loads, although it appears to correlate with strain to some degree in Figure 26.

**Table 14:** Comparison of morphological variables predicting bite strain for hi-res FEMs.

	Intercept	ln(Length)	PC1	PC2	df	logLik	AICc	ΔAICc	Akaike weight
**1**	−16.87	1.84			3	10.46	−6.93	0.00	1.00
**3**	−6.4		−0.3	−0.17	4	9.65	8.7	15.63	0.00
**2**	−6.4		−0.3		3	2.43	9.14	16.06	0.00

Morphological variables are in order with AICc-best first. Columns correspond to parameter estimates for each morphological variable, log-likelihood, of morphological variable given data, AICc scores, ΔAICc from AICc-best, and Akaike weight.

**Table 15:** Comparison of morphological variables predicting shake strain for hi-res FEMs.

	Intercept	ln(Length)	ln(Symph. L.)	Ln(Angle)	PC1	PC2	df	logLik	AICc	ΔAICc	Akaike weight
**4**	−7.07				−0.31		3	3.14	7.71	0.00	0.51
**2**	−9.28		0.53				3	3.06	7.88	0.17	0.47
**1**	−14.06	1.23					3	−0.09	14.18	6.47	0.02
**3**	−8.88			0.72			3	−1.19	16.38	8.66	0.01
**5**	−7.07				−0.31	0.03	4	3.27	21.45	13.74	0.00

Morphological variables are in order with AICc-best first. Columns correspond to parameter estimates for each morphological variable, log-likelihood, of morphological variable given data, AICc scores, ΔAICc from AICc-best, and Akaike weight.

**Table 16:** Comparison of morphological variables predicting twist strain for hi-res FEMs.

	Intercept	ln(Length)	ln(Symph. L.)	Ln(Angle)	PC1	PC2	df	logLik	AICc	ΔAICc	Akaike weight
**2**	−10.52		0.66				3	5.52	2.96	0.00	0.57
**1**	−19.25	2.02					3	4.67	4.66	1.69	0.24
**4**	−7.77				−0.37		3	3.92	6.16	3.19	0.12
**5**	−7.77				−0.37	−0.14	4	10.46	7.07	4.11	0.07
**3**	−8.28			0.2			3	−2.94	19.88	16.92	0.00

Morphological variables are in order with AICc-best first. Columns correspond to parameter estimates for each morphological variable, log-likelihood, of morphological variable given data, AICc scores, ΔAICc from AICc-best, and Akaike weight.

The AICc-best EM of shake strain was the first principal component from the geometric morphometric analysis (PC1) (Table 15). This axis separates *T. schlegelii* with its very narrow mandible from the more robust mandibles of *C. moreletii* and *O. tetraspis*. The next best EM is virtually identical to the AICc-best (ΔAICc 0.17) has symphyseal length as the sole predictor. The explanatory model with the eigenscores from both PC1 and PC2 was the worst of all explanatory models shake strain (ΔAICc 13.74).

For twist strain, the AICc-best explanatory model had symphyseal length as the sole predictor (Table 16). The next best EM was similarly informative (ΔAICc 1.69), with Length as the sole predictor. The third and fourth EMs with PC1 alone and combined PC1 and PC2 as predictors respectively were also potentially informative (ΔAICc 3.19 and 4.11 respectively), though these are not as good as the first two EMs. Regardless, angle was a very poor explanatory model of twist strain (ΔAICc 16.92).

**Figure 27:**
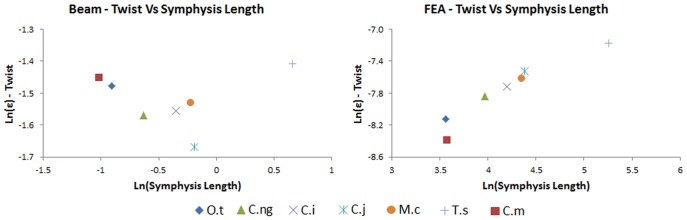
Peak strain under twist loads for beam and FE models. Peak strain under twist loads plotted against symphyseal length for beam (left) and FE (right) models. Note the relationship between symphyseal length and strain predicted by beam models is inverted in the complex FE models; additionally, beam models fail to predict ranked order under twisting. Data is plotted as natural logarithms of linear measurements (mm).Taxon abbreviations are as follows: O.t, *Osteolaemus tetraspis*; C.ng, *Crocodylus novaeguineae*; C.i, *Crocodylus intermedius*; C.j, *Crocodylus johnstoni*; M.c, *Mecistops cataphractus*; T.s, *Tomistoma schlegelii*; C.m, *Crocodylus moreletii*.

Qualitative comparison of Beam and FE models shows that beam models accurately predict ranked performance under *biting*, partially predict rank under *shaking*, and completely fail to predict rank under *twisting* (Table 17). Under *twisting*, the relationship between Symphyseal Length measurements and strain predicted by beam models was inverted in the complex FE models (Figure 27).

**Table 17:** Ranked performance of beam and FE models.

	Bite		Shake		Twist	
Rank	Beam	FEA	Beam	FEA	Beam	FEA
**1**	*Cm*	*Cm*	*Cm*	*Cng*	*Cj*	*Cm*
**2**	*Ot*	*Ot*	*Ot*	*Cm*	*Cng*	*Ot*
**3**	*Cng*	*Cng*	*Cng*	*Ot*	*Ci*	*Cng*
**4**	*Mc*	*Mc*	*Ci*	*Ci*	*Mc*	*Ci*
**5**	*Ci*	*Ci*	*Mc*	*Cj*	*Ot*	*Cc*
**6**	*Ts*	*Cj*	*Cj*	*Mc*	*Cm*	*Cj*
**7**	*Cj*	*Ts*	*Ts*	*Ts*	*Ts*	*Ts*

Performance of FEA models is assessed by the 95% strain values for *biting*, *shaking*, and *twisting.*

Taxon abbreviations are as follows: *Ot, Osteolaemus tetraspis; Cm, Crocodylus moreletii; Cng, Crocodylus novaeguineae; Ci, Crocodylus intermedius; Cj, Crocodylus johnstoni; Mc, Mecistops cataphractus; Ts, Tomistoma schlegelii*.

## Interpretation and Discussion

### Symphyseal Length in Mandibular Mechanics

The results show that symphyseal length is an important aspect of shape in determining the mechanical response of the crocodilian mandible to *shaking* and *twisting* loads (Hypothesis B). This correlation is consistent with ‘armchair’ predictions (argument from first principles) based upon beam theory, and is partly consistent with beam modelling. AICc explanatory model selection indicates that symphyseal length is the best simple measurement at predicting mandibular strain under these loads, and is even better than a multivariate measure of shape (PC1 score) for *twisting* loads. PC1 eigenscore is a slightly better predictor of strain than symphyseal length in *shaking* loads, although it should be noted that symphyseal length is a large component of the shape variation associated with PC1. Length was also an effective predictor of strain under *twisting* loads, and also covaries with symphyseal length. As *twisting* and *shaking* behaviours are used by crocodilians to feed on large prey, these results provide direct correlations between simple morphological variables and feeding ecology.

### Biting

**Figure 28:**
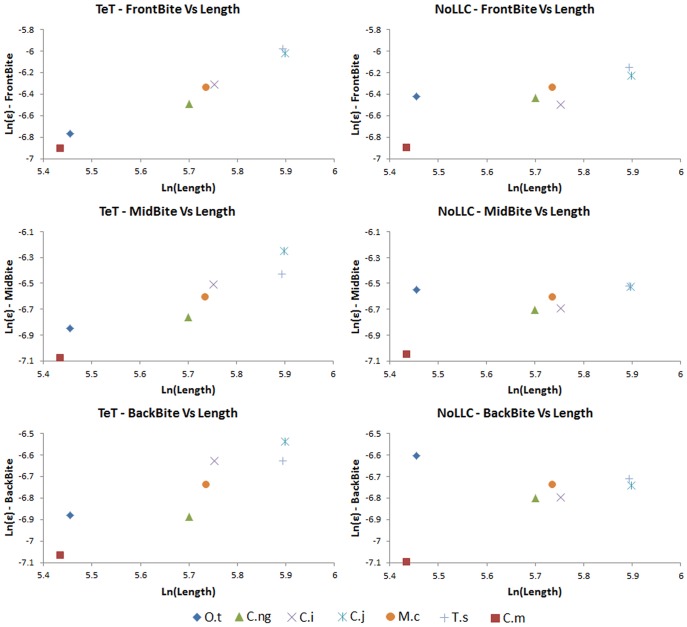
Strain in biting loads for TeT and NoLLC. Left: Strain response of mandibles when subject to equal bite force (TeT), plotted against length for (from top) front, mid and back bites. Right: Strain response of mandibles at maximal bite force (NoLLC), plotted against length for (from top) front, mid and back bites. In the TeT load cases, muscle forces are adjusted so that all models experience the same bite force as the *M. cataphractus* model for each bite point; with the exception of the *Osteolaemus* model, this has little effect on the qualitative pattern of results, with longirostrine taxa exhibiting higher strain in TeT and NoLLC load cases. Data is plotted as natural logarithms of linear measurements (mm).

Also consistent with armchair predictions and beam modelling, length was the most important determinant of mechanical strain under *biting* loads (Hypothesis B). Length is a much better predictor of strain than any other linear variable, and is also a much better predictor than multivariate measurements of shape (PC1) (Hypothesis C). In *biting*, mandibular strain is higher in longirostrine crocodiles, both when bite force is standardised (TeT) and when bite simulates maximum muscle force (‘volume scaled’; Figure 28). In the latter case bite force in longirostrine forms decreases as outlever length increases, so the higher strain may indicate a more gracile mandible in these forms in addition to the effects of increased bending moments acting on the jaws.

**Figure 29:**
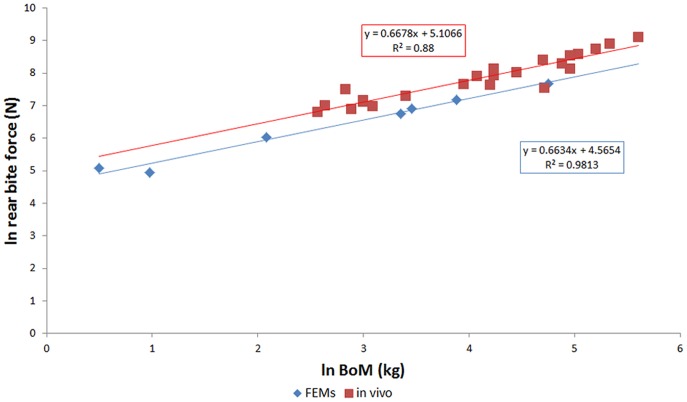
Comparison of FEM predictions and *in vivo* measurements of bite force. Natural logarithms of FEM predicted bite force (red squares) and *in vivo* bite force (blue diamonds), plotted against body mass. Bite force is for rear bites, *in vivo* bite force data from Erickson [47]. For the FEMs, body mass was calculated from skull volume using the equation *log10 body mass = log10 (skull volume x 0.9336+1.9763)* using data from McHenry [29]. Slopes of regression lines are similar, but the difference in intercept means that the *in vivo* bite force is a factor of approximately 1.6 times the FEM predicted bite force for crocodilians of a given mass.

Relative bite forces accord with *in vivo* studies of crocodilians, although absolute simulated forces are lower. Predicted bite force was consistent between volume scaled FEMs, correlating with outlever length. Given the marked variation in cranial morphology between the models, this result is consistent with the recent finding by Erickson and colleagues [47] that, for a particular bite point, bite force in crocodilians is controlled by body size rather than skull morphology (Figure 29). The absolute bite force predicted by the FEMs is consistently and significantly less than empirical data reported by [47]. The discrepancy is most likely because the jaw muscles in the FEMs are modelled as parallel fibred beams that run as straight lines between attachment points, whilst crocodilian muscles are actually pennated and run around bony structures (for example, M. pterygoidius posterior, which wraps around the ventral surface of the angular), aspects that are expected to increase total muscle force and effective inlever length. Specific tension of jaw muscles is not often measured in reptiles but in *Sphenodon punctatus* is 89 KPa [49], a figure that is much greater than isometric values used in our models (30 KPa: [15]), and this may be a source of error which may also contribute to differences in bite force between our results and experimental findings [47]. Whilst rostral proportions vary markedly between crocodilian taxa (Figure 2), the morphology of the postorbital region and adductor chamber is conservative [50], [51] and since this region houses the adductor musculature it is likely that, size for size, crocodilians of different species produce similar maximal jaw muscle force [47]. As we calculated jaw muscle forces from the osteology of the adductor chamber, the qualitative patterns of bite force predicted by the FEMs appear to be consistent with the empirical data, even if absolute force magnitude is less.

If maximal jaw adductor muscle force in longirostrine crocodiles is similar to that of mesorostrine forms, but strain under a given load is higher, then longirostrine crocodiles should be expected to avoid dangerously high strain by either having high safety factors (so that even maximal bite force will not damage bone), or by voluntarily limiting bite force. Note that the suggestion that safety factors in crocodilian skulls are high is inconsistent with *in vivo* strain data from rostra in *Alligator mississippiensis* [52], and stresses in crocodilian teeth [47].

### Beam Modelling vs ‘armchair’ Predictions

The results from beam modelling are consistent with the argument from beam theory for *biting* and *shaking*; for the former, strain will be determined by length, but for the latter strain will be determined by symphyseal length (Hypothesis A). Under *twisting*, however, the beam models found inter-rami angle, not symphyseal length, to be the best predictor of strain.

### Beam Modelling vs Complex FEMs

Both sets of beam models indicate that strain in *biting*, *shaking*, and *twisting* can be predicted from measurements of length, symphyseal length, and angle respectively. Whilst the results from the complex FEMs were consistent with these predictions for *biting* and shanking, inter-rami angle did not correlate with strain in the FEMs was an extremely poor explanatory model according to AICc-based selection. This constitutes an important discrepancy between the beam and complex FE models (Hypothesis C).

Another important comparison is the qualitative predictions of beam models vs complex FEMs. In *biting*, the beam models ranked relative performance of the mandibles the same as the FE models; this result means that in order to correctly rank the biomechanical performance of the seven mandibles tested here under *biting* loads, the only information required is mandible length. For *shaking*, beam and complex FE models agreed on the relative performance of five models but differed in their rankings of the *M. cataphractus* and *C. novaeguineae* models. For *twisting*, ranked results were similar for only four models.

The largest discrepancy between the beam modelling and FE modelling is for twist loads; the beam models found angle to be the best predictor of strain, whilst the complex FEMs found symphyseal length as the best sole predictor. This result may indicate the limitations of modelling a complex shape such as a crocodilian mandible as a beam. However, the beam models used here were very simple so it is possible that a very small increase in their complexity (such as allowing beam section to vary along the length of the beam, especially in the vertical axis) may capture an important aspect of the actual 3-dimensional structure and improve the predictive power of the beam models compared with the complex FE models.

### Functional Interpretation - Crocodilians

Ultimately, we are interested in the biomechanics that influence mandibular morphology in crocodilians. Whilst torsional loads (which are moments) cannot be directly compared to forces, the response of the mandible to biting and shaking loads can be compared. In all taxa except *Osteolaemus* the mandible is stronger under shaking loads than under equivalent biting loads (Figure 25). If, in an evolutionary sense, symphyseal length is controlled by *shaking* and *twisting* behaviours, we might expect that these behaviours should result in strain values that are at least of the same order as the strain resulting from *biting*. When shake forces were equalised to bite forces, the mandible was weaker in *biting* than in *shaking* for all species except *Osteolaemus.* For the loads used in this study, strain was higher in *biting* for all species modelled than in *shaking*, and strains resulting from *twisting* were much lower. If these loads accurately represent the magnitudes of loads used by crocodiles, then our results suggest that selection should result in increased resistance to bending loads from *biting*, rather than *shaking* or *twisting*, as a mandible that is strong enough to cope with a crocodile’s own bite force is already strong enough to cope with likely *shaking* or *twisting* loads. If, however, the loads used in *shaking* and/or *twisting* are actually much higher than those used here, then *shaking* and/or *twisting* could possibly have the strongest influence on mandibular morphology, resulting in a morphology that is stronger under these loads than in biting. Whilst we currently lack the quantitative data required to explore this aspect further, these data are in theory accessible from studies of crocodilian behaviour and as such will provide insight into the behaviours that have determined the evolution of skull form in crocodilians.

Although structural modelling can identify the biomechanical advantages of a short mandibular symphysis, the question of why longirostrine crocodilians have an elongate symphysis remains open. Clearly, it is not for increased strength, though an elongate symphysis might offer hydrodynamic advantages for rapid jaw closure during capture, or allow greater acceleration of the jaws towards agile prey. To address this question, a combination of *in vivo* data, fluid dynamics and solid mechanics would be required to best model crocodilian jaws during prey capture.

### Functional Interpretation - other Taxa and Palaeobiology

For palaeobiologists, one of the interesting aspects of crocodilians is their potential to act as an extant model for cranial palaeobiomechanics of various fossil groups which have superficially similar morphologies such as plesiosaurians, ichthyosaurians, phytosaurs, and of course extinct crocodylomorphs [8], [23], [53]. Although overall head shape may be similar between these groups, the details of skull anatomy are specific to each group. If the details of cranial anatomy are critical to modelling its biomechanics, then the principles elucidated from one group should not be generalised to another. However, if a small number of simple measurements can provide insight into the biomechanics of that group, then those insights may be generalised to the other groups. The results here are somewhat encouraging for palaeobiomechanists; since simple measures of mandibular shape (length and symphyseal length) provide some insight into the mechanics of the mandible, the same measurements may be applicable to all of the above fossil reptile groups, and to marine mammals such as odontocetes, archaeocetes, and basal mysticetes [54], providing an answer to the functional morphologist’s question – where to put the callipers? The next step in better understanding the functional morphology of the mandible is to quantify the relationship between shape and diet. Odontocetes may provide a suitable study group for this, given the diversity, morphological variation, and available ecological data for this important group of pelagic predators.

### Methodological Approaches

Since the early 2000s complex Finite Element models have been increasingly used to investigate skull mechanics in various fossil and living species; whilst different studies have made use of deductive and experimental approaches [17], many have used a comparative biomechanical approach to reconstruct the palaeobiology of extinct species [26], [28], [33], [55], [56], [57], [58]. Whilst comparative approaches are of high value to palaeobiology, they tend to use *post hoc* analysis and are sometimes difficult to conduct in a way that explicitly tests hypotheses of form and function. Studies that predict the mechanical consequence of specific morphologies are rarer, because of the difficulty in applying a fundamental theorem (such as beam theory) to complex shapes. By combining predictions based in beam theory with data from complex FE modelling, we are able to test *a priori* hypotheses of the mechanical consequences of changes in morphology. Some previous studies have combined beam theory with FE modelling [8], [59], but used very low resolution FE models. We here assume that the high resolution models used in the present study do capture the actual mechanics of the biological structures under study, but the models have yet to be validated and this remains an important step for future work and limitation of the present study.

### Conclusions

Longirostrine crocodilians experience higher strain than those of meso−/brevirostrine forms when subject to equivalent *biting*, *shaking* and torsional loads. In the mandible, strain in *biting* and *twisting* was best predicted by overall length and symphyseal length respectively, while *shaking* was best predicted by both symphyseal length and multivariate measure of shape (PC1). For *biting* and *twisting* simple linear measurements of the mandible provide better predictors of mechanics than a multivariate measure of shape (PC1); with overall length and symphyseal length outperforming PC1 for *biting* and *twisting* respectively.

For *biting* and *shaking*, the pattern of variation between species is consistent with predictions from beam theory, where overall length and symphyseal length are the best predictive measures of *biting* and *shaking* respectively. The response to *twisting* is best predicted by symphyseal length, while beam models predicted inter-rami angle. This divergence could be due to the exclusion of sectional variance in beam models; since beam models had uniform section and real mandibles vary their section with length, this difference could be expected to change the mechanics.

Of the hypotheses we sought to test, we found support for Hypothesis A, that strain in beam models should correlate best with length in *biting* but symphyseal length in *shaking* and *twisting*, and Hypothesis B of the same correlations in complex FE models. We found partial support for Hypothesis C, that the morphological variables that best explain strain in beam models will also best explain strain in complex FE models; this was the case under *biting* and *shaking* loads, but was not the case for *twisting* loads.

Beam theory remains a useful tool for exploring biomechanics and our approach illustrates the possibility of moving away from *post hoc* examinations of complex models towards *a priori* predictions of the fundamental mechanics. Our approach allows researchers to focus on using information from first principles to identify the components of shape that are of interest and then quantify and compare the relative statistical performance of various hypotheses using model selection criteria, something that is rarely done in current studies of biomechanics.
